# Septin11 promotes hepatocellular carcinoma cell motility by activating RhoA to regulate cytoskeleton and cell adhesion

**DOI:** 10.1038/s41419-023-05726-y

**Published:** 2023-04-20

**Authors:** Lisheng Fu, Xiaoyan Wang, Ying Yang, MeiHua Chen, Adilijiang Kuerban, Haojie Liu, Yiwei Dong, QianQian Cai, Mingzhe Ma, XingZhong Wu

**Affiliations:** 1 Department of Biochemistry and Molecular Biology, School of Basic Medical Sciences, and Department of Cardiology of Huadong Hospital Affiliated to Fudan University, Fudan University, 200032 Shanghai, People’s Republic of China; 2 Key Laboratory of Medical Molecular Virology (MOE/NHC/CAMS), Microbiology and Parasitology, School of Basic Medical Sciences, Shanghai Medical College, Fudan University, 200032 Shanghai, People’s Republic of China; 3 NHC Key Laboratory of Glycoconjugates, Department of Biochemistry and Molecular Biology, School of Basic Medical Sciences, Fudan University, 200032 Shanghai, People’s Republic of China; 4 Department of Cardiology, Huadong Hospital Affiliated to Fudan University, Fudan University, 200040 Shanghai, People’s Republic of China; 5 Shanghai Key Laboratory of Molecular Imaging, Shanghai University of Medicine and Health Sciences, 201318 Shanghai, China; 6 Department of Gastric Surgery, Shanghai Cancer Center of Fudan University, 200032 Shanghai, People’s Republic of China

**Keywords:** Tumour biomarkers, Oncogenes, Focal adhesion

## Abstract

Septins as GTPases in the cytoskeleton, are linked to a broad spectrum of cellular functions, including cell migration and the progression of hepatocellular carcinoma (HCC). However, roles of SEPT11, the new member of septin, have been hardly understood in HCC. In the study, the clinical significance and biological function of SEPT11 in HCC was explored. SEPT11 was screened out by combining ATAC-seq with mRNA-seq. Role of SEPT11 in HCC was further investigated by using overexpression, shRNA and CRISPR/Cas9-mediated SEPT11-knockout cells or in vivo models. We found RNA-seq and ATAC-seq highlights LncRNA AY927503 (AY) induced SEPT11 transcription, resulting in Rho GTPase activation and cytoskeleton actin aggregation. The GTP-binding protein SEPT11 is thus considered, as a downstream factor of AY, highly expressed in various tumors, including HCC, and associated with poor prognosis of the patients. In vitro, SEPT11 overexpression promotes the migration and invasion of HCC cells, while SEPT11-knockout inhibits migration and invasion. In vivo, SEPT11-overexpressed HCC cells show high metastasis incidents but don’t significantly affect proliferation. Meanwhile, we found SEPT11 targets RhoA, thereby regulating cytoskeleton rearrangement and abnormal cell adhesion through ROCK1/cofilin and FAK/paxillin signaling pathways, promoting invasion and migration of HCC. Further, we found SEPT11 facilitates the binding of GEF-H1 to RhoA, which enhances the activity of RhoA. Overall, our study confirmed function of SEPT11 in promoting metastasis in HCC, and preliminarily explored its related molecular mechanism. SEPT11 acts as an oncogene in HCC, also draws further interest regarding its clinical application as a potential therapeutic target.

## Introduction

Hepatocellular carcinoma (HCC) accounts for 90% of the cases of primary liver cancer and is the leading cause of cancer-related death worldwide [1]. At present, most HCC patients are often accompanied by regional spread and metastasis, and the prognosis of patients is poor [2, 3]. Therefore, there is an urgent need to investigate the cellular biology and molecular mechanisms of HCC metastasis, providing opportunities for improving clinical outcomes and developing better treatment options.

Long non-coding RNAs (LncRNAs) are closely associated with the progression of cancer. Hundreds of HCC-related LncRNAs regulate gene expression through different patterns, thereby exerting the function as oncogenes or tumor suppressor genes in HCC [4]. Our previous study has explored the function of AY as an oncogene in the progression of HCC [5], but the downstream regulatory signals of its function were unclear.

The development of tumor cells that acquire metastatic potential into a more motile and invasive phenotype is a critical step in tumor metastasis, and these processes require cytoskeleton rearrangement and abnormal cell adhesion [6, 7]. Many evidences suggest that some Rho GTPases, especially RhoA, Rac1 and Cdc42, are key regulators of the process [8, 9]. Rho GTPases are mainly involved in cell morphological changes, cell adhesion and regulation of cytoskeleton reorganization, which are essential for invasion and migration of tumor cells [10]. According to reports, LncRNAs can regulate the cytoskeleton directly or may influence the cytoskeleton via Rho/ROCK signaling during tumor migration [11].

Septins are highly conserved cytoskeletal GTPases, closely associated with actin, microtubule and associated motion. Recently, septins have been identified as the fourth major component of the cytoskeleton, involved in major membrane motility processes such as cytokinesis, vesicle trafficking and exocytosis [12, 13]. Various septins have been reported to play a role in cancer progression [14–16]. SEPT11 has been found to co-localize with microtubules and actin stress fibers to regulate cytoskeleton [17]. It has also been reported that SEPT11 may be associated with the progression of differentiated thyroid cancer and acute lymphoblastic leukemia [18, 19]. As a new member of the septin family, SEPT11 presumably plays an important role in tumor progression like other septins, but there has not been reported about its function in HCC.

In summary, we reported the function of SEPT11 in HCC for the first time. We also found that the expression of SEPT11 is regulated by LncAY, and high expression of SEPT11 in HCC predictes a poor clinical prognosis and promoted the metastasis of HCC. Interestingly, the effect of SEPT11 on cell proliferation is not significant. In addition, we preliminarily explored the mechanism of SEPT11 promoting cell migration and invasion. SEPT11 targets RhoA in vitro, regulates cytoskeleton rearrangement and abnormal cell adhesion through ROCK1 and FAK signaling pathways, thereby promoting the invasion and migration of HCC. The findings of this study demonstrate that SEPT11 acts as an oncogene in HCC and is expected to become a new therapeutic target for HCC.

## Materials and methods

### Patients samples

The tumor and matched peritumoral samples were obtained from 76 patients with HCC, which was diagnosed by the Tumor Hospital Affiliated to Fudan University. All studies involving human samples have been approved by Fudan Biomedical Ethics Committee (Approval No.14000000020000027). Patients’ clinicopathological characteristics and other relevant clinicopathological data were from hospital records (Supplementary Table 1). Patients’ additional personal information was protected.

### Cell culture and transfection

Hep3B, HepG2, LM3, 7721, Huh7, and HEK-293T cells were from Cell Bank of Type Culture Collection of Shanghai Institute of Biochemistry & Cell Biology. Human hepatocytes THLE-2 cell was obtained from ATCC, purchased from Qingqi (Shanghai, China) Biotechnology Development Co., Ltd. These cells cultured in Dulbecco’s Modified Eagle’s Medium (Gibco-Life Technologies) supplemented with 10% fetal bovine serum (FBS), the growing of THLE-2 cell need to supplement with 5 ng/mL EGF, 70 ng/mL phosphoethanolamine. Hep3B, HepG2, Huh7, LM3, HEK-293T and THLE-2 cells were identified by STR (short tandem repeats). The 7721 cells were identified by their morphological characteristics. All cells were cultured in a cell incubator with 5% CO_2_ at 37 °C. Cells were transfected with plasmid DNA at about 70–80% confluence, and 6 μg of plasmid was transfected into each 6 cm dish. Rhosin inhibitor for RhoA (30 μM), Y27632 for ROCK (10 μM) and PF-573228 for FAK (10 μM) were purchased from MedChemExpress (MCE, USA).

### Stable transfection cell lines and SEPT11 knock-out cells

Stable cell lines for AY overexpression and shAY were preserved in our laboratory. Lentiviral production, cell infection and selection were performed as previously described [20]. Briefly, the SEPT11 overexpression plasmid or the lentiCRISPR-v2 vector inserted with sgRNA (KO-SEPT11 Oligo: CACCGGTCAACAAGTCTACTTCTCA, Lacz-KO Oligo: TGCGAATACGCCCACGCGATGGG) was mixed with the packaging plasmid psPAX2 (Addgene, 12260), and the envelope plasmid pMD2.G (Addgene, 12259) were mixed, then transfected into 293T cells. After 48 h, the virus in the supernatant was collected. Cells were infected with virus and polybrene (MCE, HY-112735) was added at a final concentration of 8 μg/ml. After 48 h, the corresponding resistance medium was added to screen. HepG2 cells were added with 2 μg/ml of G418 (Sigma, A1720) or 1 μg/ml of puromycin (MCE, HY-B1743A), while Huh7-luciferase cells were added with 5 μg/ml of G418 or 2 μg/ml of puromycin to screen the cells with stable expression of related genes. The SEPT11 overexpression and the SEPT11 knock-out cells were propagated in complete medium including puromycin.

The cells were limiting diluted to a 96-well plate (~30 cells/plate). After 1 to 2 weeks, the clones were picked, and the genomic DNA was extracted by Quick Extract isolation DNA Extraction Solution (Lucigen, QE0905T). The DNA was used for PCR, then PCR products were recycled and sequenced. Monoclonal cell lines proteins were also extracted for WB.

### Animal studies

The 5-week-old female BALB/C nude mice were reared and managed by the SPF Experimental Animal Center of Fudan University, and were purchased from Shanghai SLAC company. All animal experiments were approved by the Animal Ethics Committee, School of Basic Medical Sciences, Fudan University (permit number 20140226-001). The animals were randomly assigned, 6 animals per group. The experimental conception and design have been done by the investigators, but the investigators were blinded to the group allocation during assessing the results. Stable HepG2 or Huh7-luciferase cells were resuspended in serum-free Dulbecco’s modified Eagle medium (DMEM) medium at a concentration of 1 × 10^7^/ml (mice injected subcutaneously) or 1 × 10^5^/ml (mice systemic circulation injected via tail vein), while mice injected subcutaneously the HepG2 cells suspension added to Matrigel (Corning, 354234). According to the manufacturer’s instructions, Rhosin (30 mg/kg) was injected intraperitoneally into nude mice twice a week. After 28 days, mice were observed and photographed by mouse in vivo fluorescence imaging system, while mice were injected with HepG2 cells were sacrificed and tumor tissues were taken out, then photographed and observed the tumors, livers and lungs. These tissues were fixed, then prepared tissue sections

### ATAC-seq and mRNA-seq

ATAC-seq and mRNA-seq service was provided by CloudSeq Biotech (Shanghai, China). RNA libraries were constructed using the Illumina TruSeq Stranded Total RNA Library Prep Kit (Cat. #RS122-2301). The mRNA-seq was performed on Illumina HiSeq instrument using 150 bp paired-ended mode, then the sequencing and computational analysis was as previously published [21]. For ATAC-seq, nuclei were extracted and processed as previously described [22]. We required a minimum of 10 million aligned reads after removal of duplicate and non-uniquely aligning reads. GO and pathway enrichment analysis was performed based on the differentially expressed mRNAs.

### Bioinformatics analysis

Genotype-tissue expression (GTEx) database (https://gtexportal.org/) to obtain the gene expression profiling in 31 tissues. From Cancer Cell Line Encyclopedia (CCLE) database (https://portals.broadinstitute.org/ccle/), we downloaded gene expression data in 21 tissue-derived tumor cell lines. The Cancer Genome Atlas Liver Hepatocellular Carcinoma (TCGA-LIHC) data is publicly available from the University of California, Santa Cruz (UCSC) Genome Browser (https://xenabrowser.net) [23]. We performed Gene Set Enrichment Analysis (GSEA) on differences in KEGG pathway enrichment between low SEPT11 and high SEPT11 HCC samples from the TCGA.

### Immunoprecipitation and western blotting

The assays were performed as described previously [5, 24]. Briefly, HCC cells or tissue were lysed in cell lysis buffer for Western and IP (Beyotime, P0013) with phosphatase inhibitor cocktail (Beyotime, P1082). For immunoprecipitation, protein A/G agarose beads (Beyotime, P2012) coupled with primary antibodies. After 1 h of rotation (4 °C), beads were washed three times in lysis buffer, then the beads were added to cell lysates with cocktail. After 8 h of rotation (4 °C), beads were washed three times, then 2× SDS loading were added and boiled for 5 min. The prepared protein samples were run on 10% or 15% polyacrylamide-SDS gels, then transferred onto PVDF membranes. Next, membranes were sealed in 3% BSA for 1 h, washed three times in TBST buffer. The membranes incubated with the primary antibodies for 12–15 h (4 °C). Hereafter, membranes were incubated with secondary antibody and signals were visualized by ECL.

The following antibodies were used: Anti-GAPDH (Proteintech, 60004-1-Ig); Anti-Actin antibody (Millipore, MAB1501); Anti-Cdc42 antibody (Cytoskeleton, ACD03); Anti-Rac1 antibody (Cytoskeleton, ARC03); Anti-RAC1/2/3 antibody (Proteintech, 16117-1-AP); Anti-RhoA antibody (Cytoskeleton, ARH05); Anti-RhoA antibody (Proteintech, 10749-1-AP); Anti-RhoB antibody (Proteintech, 14326-1-AP); Anti-RhoC antibody (Proteintech, 10632-1-AP); Anti-SEPT11 antibody (Proteintech, 14672-1-AP); Anti-PCNA antibody (Proteintech, 10205-2-AP); Anti-Cyclin D1 (Proteintech, 60186-1-Ig); Anti-ROCK1 antibody (Proteintech, 21850-1-AP); Anti-ROCK2 antibody (Proteintech, 21645-1-AP); Anti-LIMK1 antibody (Proteintech, 19699-1-AP); Anti-GEF-H1 antibody (Proteintech, 24472-1-AP); Phospho-GEF-H1 (S886) antibody (CST, 14143); Anti-FLAG-Tag antibody (CST, 8146); Anti-HA-Tag antibody (CST, 3724); Anti-LIMK1 antibody (Abmart, TA6345); Phospho-LIMK1 (T508) antibody (Abmart, TP56325); Anti-FAK antibody (Abmart, T55464); Phospho-FAK (Y397) antibody (Abmart, TA3398); Anti-paxillin antibody (Abmart, T55274); Phospho-FAK (Y118) antibody (Abmart, T55891); Anti-Src antibody (Abmart, T56605); Phospho-FAK (Y416) antibody (ABclonal, AP0452); Anti-Cofilin antibody (Cohesion, CPA4598); Phospho-Cofilin (S3) antibody (Cohesion, CPA1625); Anti-IQGAP1 antibody (Cohesion, CPA2326).

### The ratio of F-actin to G-actin

The F-actin to G-actin ratio was performed as described previously [25]. Briefly, F-actin is insoluble, whereas G-actin is soluble. According to the difference, cells were lysed in cold lysis buffer, then centrifuged at 13,000 × *g* for 30 min. G-actin was in the supernatant, while F-actin in the precipitate was resuspended in lysis buffer, added the same volume of lysis buffer and incubated at 4 °C for 1 h to convert F-actin into G-actin. Then, the liquid was centrifuged at 13,000 × *g* for 30 min, and saved the supernatant. The F-actin to G-actin ratio was determined by western blotting using a specific actin antibody.

### Immunofluorescence and confocal microscopy

The assays were performed as described previously [26]. Briefly, Cells were seeded on sterile cover slips for 24 h, then fixed for 10 min in 4% paraformaldehyde at room temperature. Cells perforated with 0.5% Triton for 15 min and blocked with 3% BSA for 30 min. The primary antibody or/and Phalloidin-TRITC (Sigma, P1951) diluted by 3% BSA was added and incubated at 37 °C for 2 h. The second antibody diluted by 3% BSA was added and incubated at 37 °C for 1 h. Cells stained with DAPI for 2 min. Images were acquired using the confocal laser scanning microscope (Leica TCS SP8) at ×600 magnification, and the quantification of fluorescence was evaluated with ImageJ.

### Rho GTPase activity assay

Rho GTPases activity was measured using the RhoA/Rac1/Cdc42 activation assay biochem Kit™ (Cytoskeleton), according to the manufacturer’s instructions. Briefly, cells were lysed in cold lysis buffer. 300–800 μg of cellular extracts were incubated with 10–15 μg Rhotekin-RBD or PAK-PBD affinity beads. After 1 h of rotation (4 °C), the beads were washed, then added 2× Laemmli buffer and been boiled. Pulled-down RhoA/Rac1/Cdc42 was detected by immunoblotting, and normalized to total RhoA.

### Cell migration, invasion, wound healing and colony formation assay

The assays were performed as described previously [5, 27]. Briefly, cell count is made to be 10^4^ cells/ml by DMEM, and 1 ml cell suspension was added in the migration and invasion chambers. DMEM complete medium was added to the inside of the chamber. After 24 h, the chambers were fixed, stained and counted.

The transfected HCC cells were cultured in 6-well plates, scratched when the density was close to 100%, then removed the scattered cells. HCC cells were maintained with 1%FBS medium, and photographed at different times.

The transfected HCC cells were suspended, then counted and pipetted approximately 500 cells into a 6-well plate. The cells were cultured in complete medium containing 10% FBS. Two weeks later, the cells formed visible clones. The clones were fixed, stained, and counted.

### Statistical analysis

Data analysis was performed using GraphPad Prism 8.0 software. Significance of groups were determined by unpaired Student’s *t*-test or one-way ANOVA for comparisons. Bonferroni’s correction or Tukey’s multiple comparisons were used to prevent error accumulation as a result of multiple hypothesis tests. Pearson’s correlation analysis was used to analyze the correlation between two indices. The correlation with expression of SEPT11 and clinicopathological parameters was performed by the Pearson *χ*^2^ test. The data were presented as mean ± S.E. or SD for bar graphs, and experiments were repeated at least three times. *p* < 0.05 was considered statistically significant.

## Results

### RNA-seq and ATAC-seq highlights LncAY induced transcription

**Fig. 1:**
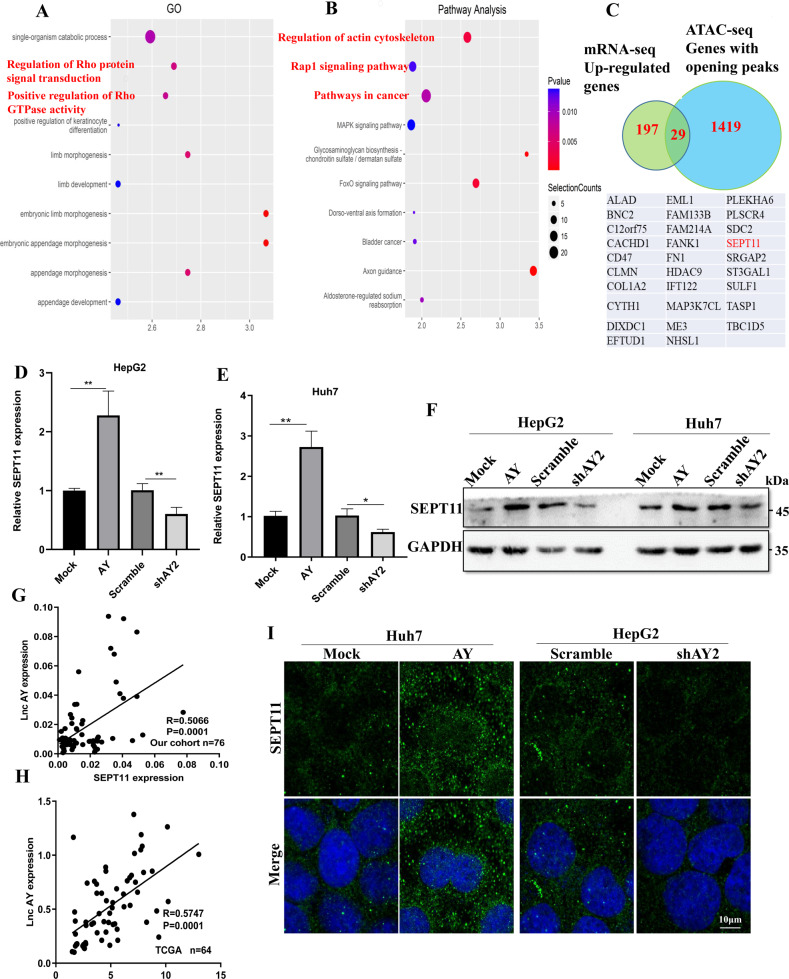
AY regulated the expression of SEPT11 in HCC cells. **A** GO analysis of genes with open sites in ATAC-seq; **B** KEGG analysis of genes with open sites in ATAC-seq; **C** aggregation of genes upregulated in mRNA-seq and with open sites in ATAC-seq; **D**, **E** After overexpression and shAY, the transcription level of SEPT11 was detected by QPCR, * means *p* < 0.05, ** means *p* < 0.01, the same below; **F** After overexpression and shAY, the protein content of SEPT11 was detected by WB; **G** after QPCR detection of 76 cases of HCC and paired paracancerous tissues, the correlation between AY and SEPT11 expression was analyzed (samples with less than 35 CT values were retained); **H** The correlation between AY and SEPT11 expression was analyzed according to the expression profile of HCC in TCGA database; **I** the expression of SEPT11 protein after overexpression and shAY was observed by cellular immunofluorescence combined with confocal microscopy.

In order to explore the pathways in which AY can promote HCC, we performed mRNA-seq and ATAC-seq using HepG2 cells overexpressing AY and control group. We analyzed the mRNA-seq results and found that the genes significantly upregulated and down-regulated after AY overexpression were 226 and 263, respectively (Supplementary Fig. 1A); ATAC-seq results revealed that the significantly chromatin opening and closing sites after AY overexpression were 1671 and 733 (Supplementary Fig. 1B); the genes upregulated by AY in mRNA-seq were enriched in pathways such as guanylate kinase activity (catalytic production of GDP), cytoskeletal adaptor activity (Supplementary Fig. 1C); the genes with open sites in ATAC-seq were enriched in pathways such as regulation of Rho protein signaling, positive regulation of Rho GTPase activity (Fig. 1A); Rho GTPase signaling mainly regulates cytoskeleton and cell adhesion, thus affects tumor invasion and migration [28]. Further analysis revealed that the genes affected by AY were indeed mainly distributed in the regulation of actin cytoskeleton, Rap1 signaling pathway and pathways in cancer (Fig. 1B; Supplementary Fig. 1D). In-depth analysis of significantly different genes, we screened out the GTPase SEPT11 (Fig. 1C), present in the cytoskeleton, which is a new member of the septin family. The function of SEPT11 is closely related to actin dynamics, adhesion and cell motility, and is also involved in the regulation of Rho protein signaling and tumor progression [29, 30]. Therefore, SEPT11 may be a key downstream factor for LncAY to promote HCC progression.

### Expression of SEPT11 in HCC cells is regulated by AY

**Fig. 2:**
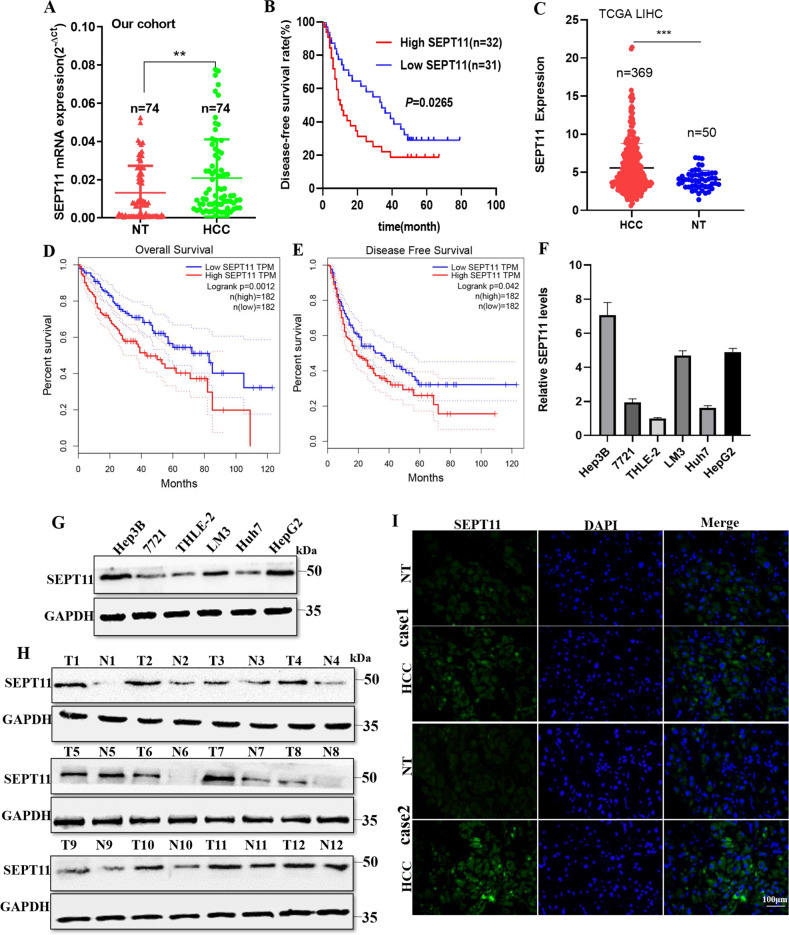
The expression of SEPT11 in HCC was detected by bioinformatics analysis and biological experiments, and its relationship with prognosis was analyzed. **A** QPCR detection of SEPT11 expression in 74 patients with HCC paired cancer and paracancerous tissues; **B** the relationship between SEPT11 expression and prognosis DFS in 74 patients with HCC; **C** The expression of SEPT11 in tumor and adjacent tissues was analyzed by TCGA database data; **D**, **E** The relationship between SEPT11 expression and prognosis of OS and DFS was analyzed by using TCGA database data; **F**, **G** QPCR and WB were used to detect the transcriptional expression and protein content of SEPT11 in hepatocytes THLE-2 and HCC cells Huh7, HCCLM3, HepG2, and Hep3B; **H** WB was used to detect the expression of SEPT11 in paired cancer and adjacent tissues (*n* = 12); **I** Immunofluorescence staining was used to observe the expression of SEPT11 in paired cancer and adjacent tissues.

The expression of endogenous SEPT11 in various HCC cell lines was tested (Fig. 2F). We selected HepG2 and Huh7 for subsequent functional related experiments, whose expression of SEPT11 was relatively high or low. In order to verify whether the expression of SEPT11 is regulated by AY, we used AY overexpression and shRNA plasmids to perform the following experiments (Supplementary Fig. 1E, F). The expression of SEPT11 was upregulated when AY was overexpressed in the two kinds of cells, while the results were reversed in shAY (Fig. 1D, E). Results of protein content detected by WB were similar to that of transcriptional expression (Fig. 1F). HCC tissues preserved in our laboratory (*n* = 76) using QPCR to detect the transcriptional expression level of AY and SEPT11 (Fig. 1G). Expression profiling of HCC in The Cancer Genome Atlas (TCGA) database (https://www.cancer.gov/) (Fig. 1H). It was found that there was a significantly positive correlated with the expression of AY and SEPT11. Further analysis showed that the expression of AY in 55 cases of HCC was higher than adjacent NT tissues, and in 49 cases of hepatocellular carcinoma with high expression of SEPT11. The 10 of 17 cases AY low-expressing HCC specimens also had low levels of SEPT11 (Supplementary Fig. 1G). Cellular immunofluorescence was observed by confocal microscopy, and it was also found that the expression of SEPT11 was regulated by AY (Fig. 1I).

### SEPT11 is significantly upregulated in HCC and associated with poor prognosis

The GTEx database was used to analyze the expression of SEPT11 in 31 tissues (Supplementary Fig. 2A). At the same time, from CCLE database, we downloaded SEPT11 gene expression data in 21 tissue-derived tumor cell lines (Supplementary Fig. 2B). We found that SEPT11 widely expresses in various tissues and tumor cell lines. Further, the expression of SEPT11 in 74 cases of HCC tissues preserved in our laboratory was also higher than that in paracancerous tissues (Fig. 2A), and was significantly associated with the prognosis of DFS (Fig. 2B). We obtained the expression of SEPT11 gene in many kinds of tumor and adjacent tissues from TCGA database and found that SEPT11 was highly expressed in various tumor tissues, including HCC (Fig. 2C; Supplementary Fig. 2C). Considering the small number of normal samples in TCGA, we integrated the data of normal tissues in the GTEx database and found similar results to the analysis of TCGA alone (Supplementary Fig. 2D).

SEPT11 expression was significantly correlated with prognosis of overall survival (OS) (Fig. 2D; Supplementary Fig. 2E) and prognosis of disease-free survival (DFS) in various tumors including HCC (Fig. 2E). At the same time, the transcriptional expression and protein content of SEPT11 in normal hepatocyte THLE-2 and HCC cell lines Huh7, LM3, HepG2, and Hep3B were detected, and SEPT11 was highly expressed in HCC cell (Fig. 2F, G). WB and immunofluorescence staining were performed on 12 paired cancer and paracancerous tissues, and it was found that SEPT11 was significantly overexpressed in cancer tissues (Fig. 2H, I). These results indicate that SEPT11 is highly expressed in HCC and is closely related to the poor prognosis.

### SEPT11 promotes cell migration and invasion in vitro

In the study, we have shown that high expression of SEPT11 in HCC is associated with poor prognosis, so what function does SEPT11 play in the progression of HCC? We investigated the relationship between SEPT11 expression and clinicopathological parameters in patients with HCC, and found that SEPT11 expression was significantly correlated with microvascular invasion and edmondson grade, but not with tumor size (Supplementary Table 2). Next, we conducted Gene Set Enrichment Analysis (GSEA) to characterize the differences between low SEPT11 and high SEPT11 HCC samples from the TCGA dataset. Of the top5 signaling pathways, four are related to cell migration (Supplementary Fig. 3A). These data suggest that SEPT11 promotes the invasion and migration of HCC cells.

**Fig. 3:**
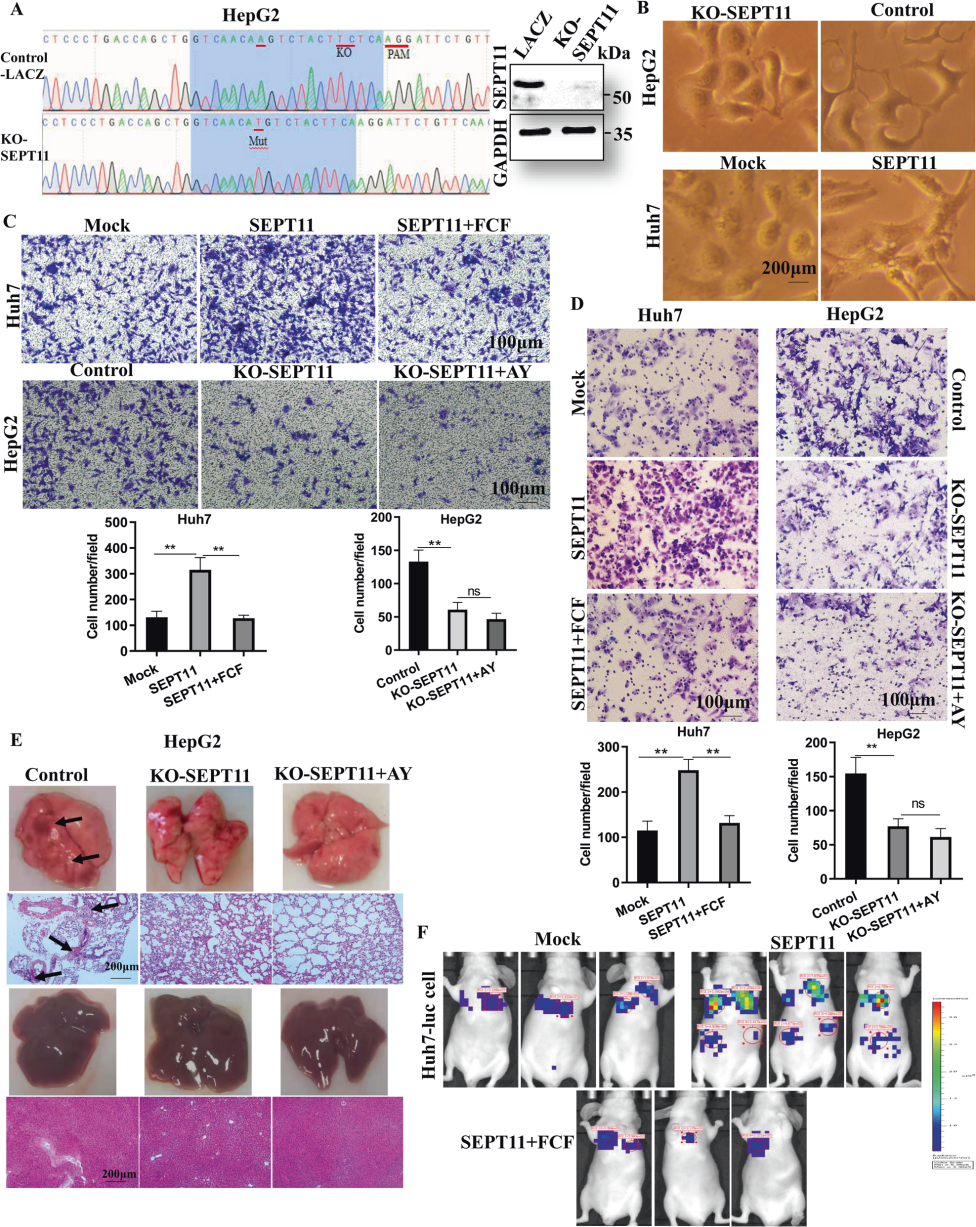
Functions of SEPT11 in HCC were analyzed by experiments in vitro and vivo. **A** Sequencing and WB to verify CRISPR/Cas9-mediated gene knockout of SEPT11 cell line; **B** Observation of the effect of SEPT11 on cell morphology; **C** Detection of the effect of SEPT11 on cell migration ability; **D** Transwell detected the effect of SEPT11 on cell invasion ability; **E** HepG2 cells were injected into the tail vein, the liver and lungs of the mice were taken for observation and photography on the 28th day, and the tissues were fixed for HE staining, the arrows indicate location of tumor focus; **F** Huh7-luciferase cells were injected into the tail vein of nude mice, observed and photographed using the mouse in vivo fluorescence imaging system on the 28th day.

In order to clarify the function of SEPT11 in HCC, we constructed a cell line of overexpression and CRISPR/Cas9-mediated gene knockout of SEPT11. SEPT11 overexpression was verified by QPCR (Supplementary Fig. 3B). The HepG2 cell line of KO-SEPT11 was screened successfully by sequencing and WB validation (Fig. 3A). Compared with the control cells, SEPT11-knockout HepG2 cells had fewer protrusions, and SEPT11 overexpression resulted in the expansion of HuH7 cells more fastly, obvious morphological changes and increased formation of filamentous pseudopodia (Fig. 3B). In addition, SEPT11 overexpression significantly promoted cell migration and invasion, while KO-SEPT11 significantly suppress the functions (Fig. 3C, D). Forchlorfenuron (FCF) can specifically inhibit the assembly of septin and its kinetics [31], and AY can promote the expression of SEPT11. We used FCF to inhibit or AY to promote SEPT11, and found that FCF could abolish the migration and invasion ability promoted by SEPT11 overexpression, while AY overexpression had little effect on KO-SEPT11 cell migration and invasion (Fig. 3C, D). Interestingly, we found that SEPT11 had no significant effect on cell proliferation (Supplementary Fig. 3C, D), and the effect of AY on the proliferation of HCC cells was not through SEPT11 (Supplementary Fig. 3C). These data confirm that SEPT11 expression is important for HCC cell motility and migration in vitro, but does not significantly affect proliferation.

### SEPT11 enhances tumor metastasis in vivo

To further explore the role of SEPT11 on HCC in vivo. The genetically engineered Huh7-luciferase cells or HepG2 cells were injected subcutaneously or into the systemic circulation (injection via tail vein) of nude mice. After 28 days, were observed and photographed by using mouse in vivo fluorescence imaging system (Fig. 3F; Supplementary Fig. 3E), while mice were injected with HepG2 cells were photographed and observed the tumors, livers and lungs, and the livers and lungs tissues were stained with HE (Fig. 3E; Supplementary Fig. 3F). No significant change in tumor size was observed in the subcutaneous tumorigenesis of the two kinds of cells treated with SEPT11 overexpression, knockout or inhibitor FCF (Supplementary Fig. 3E, F), but AY overexpression could promote tumor enlargement in KO-SEPT11 group (Supplementary Fig. 3F), this implies that AY can induce proliferation through other signals, such as the ITGAV we previously found [5]. At the same time, except for luciferase fluorescence was observed in the subcutaneous injection site of nude mice. No fluorescence was found in other parts such as liver and lung (Supplementary Fig. 3E). Mice were injected with HepG2 cells, except subcutaneous situ. No tumor nodules were found in other areas such as livers and lungs (data not shown). However, compared with the control group, the tail vein injection of Huh7-luciferase cells with overexpression of SEPT11 showed more obvious metastasis fluorescence in the lung and liver, and FCF could significantly inhibit the metastatic ability of SEPT11 (Fig. 3F). Tumor metastatic nodules were observed in the lungs of nude mice injected with HepG2 cells in the control group, but no obvious metastases nodules were observed in the lungs of KO-SEPT11 and AY + KO-SEPT11 groups, and no metastases nodules were found in livers of the three groups (Fig. 3E). Immunofluorescence CD31 staining showed obvious angiogenesis in lung tissue of nude mice injected with HepG2 cells in the control group, while the angiogenesis was weak in the KO-SEPT11 group, and AY overexpression could not rescue the reduced angiogenesis caused by KO-SEPT11 (Supplementary Fig. 3G). These data suggest that SEPT11 may promote tumor cell survival and motility in circulation, or enhance metastatic growth at secondary sites, rather than helping tumors escape from primary tumor. It also indicated that SEPT11 is an important downstream factor for AY in promoting the migration and invasion of HCC cells.

### SEPT11 promotes HCC progression by regulating of actin cytoskeleton through activated RhoA

**Fig. 4:**
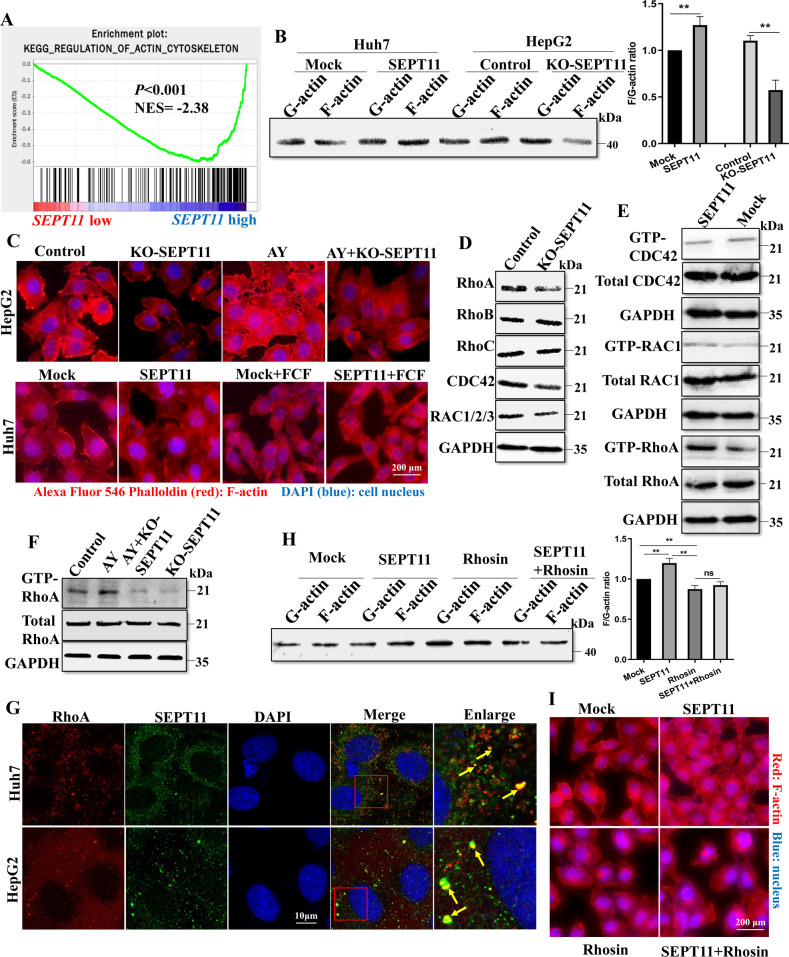
SEPT11 promotes the activity of RhoA to regulate actin cytoskeleton reorganization. **A** GSEA analysis of high expression of SEPT11 in TCGA database affects the enrichment of genes in regulatory of actin cytoskeleton pathway; **B** WB experiment detects the effect of SEPT11 expression on actin dynamics in two types of cells; **C** Cell immunofluorescence was used to detect the effect of SEPT11 on actin content and arrangement; **D** The effect of KO-SEPT11 on the content of several major Rho proteins; **E** Rho pulldown activity assay kit was used to quantify the activities of RhoA, CDC42, and RAC1 in SEPT11 overexpressing cells; **F** The effect of AY expression on the activity of RhoA; **G** The co-localization of RhoA and SEPT11 in cells was observed by immunofluorescence combined with confocal, the arrows indicate puncta of SEPT11 co-localization with RhoA; **H** WB experiment detects the effect of RhoA inhibitor Rhosin on actin dynamics; **I** The effect of RhoA inhibitor Rhosin on the content and arrangement of actin was detected by cellular immunofluorescence. **E** The effect of RhoA inhibitor Rhosin on the content and arrangement of actin was detected by cellular immunofluorescence.

The analysis of the sequencing results above suggested that the function of SEPT11 may be related to regulation of actin cytoskeleton and Rho protein signal transduction. In next analyses, GSEA results indicated that in the high SEPT11 samples were found to be enriched in gene sets related to regulation of actin cytoskeleton (*p* < 0.001) (Fig. 4A), focal adhesion and FC-gamma-r-mediated-phagocytosis (*p* < 0.001) (Supplementary Fig. 4A, B), three pathways all contain Rho proteins, which played an important role in cell migration and invasion. Meanwhile, the close connection between Septins and Rho proteins has been extensively reported [30, 32, 33], Therefore, we make a reasonable hypothesis that SEPT11 regulates the cytoskeleton to promote the progress of HCC, and this function is related to Rho protein signal.

Following the hypothesis, we examined the effect of SEPT11 on the dynamic regulation of actin in HepG2 and Huh7 cells. The remodeling of actin filament (F-actin) is an important part of cytoskeletal rearrangement, which provides the impetus for cell invasion and migration [34]. The ratio of F-actin to G-actin can reflect the balance between actin polymerization and depolymerization. The results showed that SEPT11 increased the ratio of F/G-actin, while KO-SEPT11 decreased it (Fig. 4B). At the same time, cellular immunofluorescence staining showed that overexpression of SEPT11 significantly increased actin fibers, thickened and ordered the fiber structure compared with the control, while the actin fibers decreased and disarranged in the KO-SEPT11 group. Overexpression of AY, the upstream regulator of SEPT11, significantly increased the number of actin fibers, but in the AY + KO-SEPT11 group, actin fibers did not become thickened and orderly due to the knockout of SEPT11. FCF, an inhibitor of SEPT11, reduced actin fibers and even affected cell morphology, and significantly inhibited actin fiber thickening caused by overexpression of SEPT11 (Fig. 4C). These results suggest that SEPT11 increased HCC progression by regulating of actin cytoskeleton.

Then, in KO-SEPT11 cells, we detected Rho proteins. The results showed that the contents of RhoA, CDC42 and RAC1 decreased after KO-SEPT11, the decrease of RhoA was the most obvious, and there was no significant change in RhoB and RhoC (Fig. 4D). Considering that Rho protein is activated to regulate its downstream signaling [35]. We therefore used Rho pulldown activation assay kit to quantify RhoA, CDC42 and RAC1 activity in SEPT11 overexpression cell. The results showed that SEPT11 promoted the activation of RhoA, but had little effect on CDC42 and RAC1 activity (Fig. 4E). Meanwhile, we found that AY could promote the activation of RhoA, but not in KO-SEPT11 cells (Fig. 4F). Cellular immunofluorescence observation with confocal microscopy showed that SEPT11 and RhoA were obviously co-localization in the two kinds of cells (Fig. 4G), but Co-IP did not find endogenous binding of SEPT11 to RHOA (Supplementary Fig. 4C). Next, we conducted Co-IP assays with exogenous tag plasmid to further confirm whether septin11 and RhoA have an interaction. Through the structural analysis of SEPT11, SEPT11 is consisted of three domains, including NTE (N-terminal extension), GTP binding (GTP-binding domain), and CCD (Coiled coil domain) (Supplementary Fig. 4D). We have constructed a full-length and multifunctional Flag-SEPT11 plasmid for Co-IP assay. It was found that SEPT11 did not form a firm complex with RhoA to influence its activity (Supplementary Fig. 4E). This may be due to the binding between them short-lived and relatively weak, or it works through intermediate factors. We further treated the cells with Rhosin, a specific inhibitor of RhoA, and found that it could significantly reduce the ratio of F/G-actin and abolish the increased ratio caused by overexpression of SEPT11 (Fig. 4H). At the same time, Rhosin treatment reduced the number of actin fibers and disarranged them, and abolished the phenomenon of structural thickening and ordering of actin fibers caused by overexpression of SEPT11 (Fig. 4I). These results suggest that SEPT11 increased HCC progression through activated RhoA.

### SEPT11 promotes activation of the ROCK1/LIMK/cofilin pathway, regulating cytoskeleton and migration in HCC cells

**Fig. 5:**
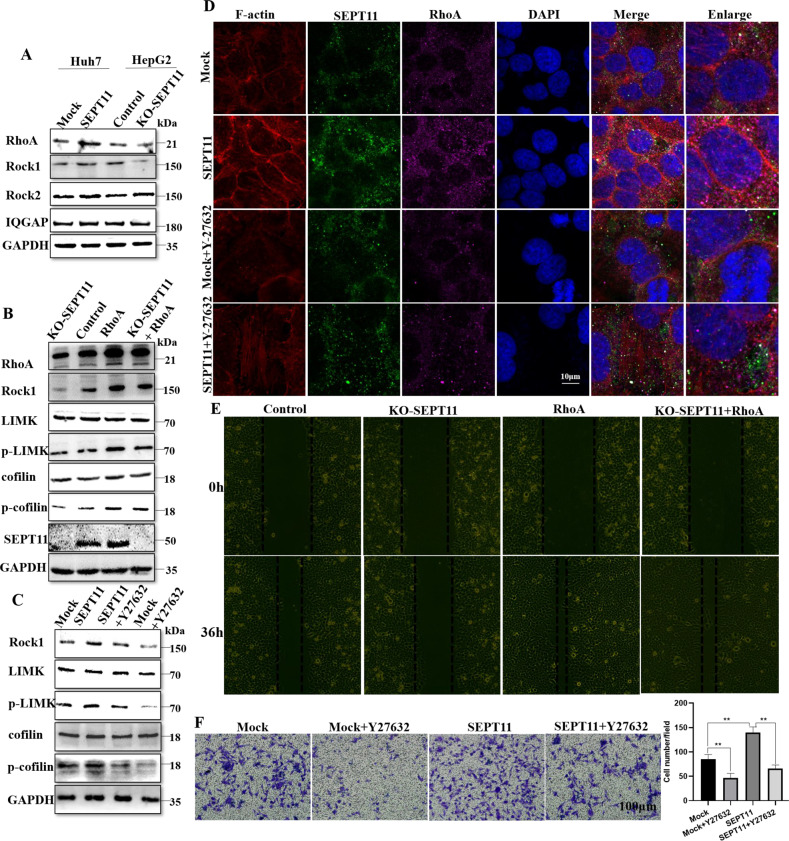
SEPT11 activates LIMK/cofilin pathway to promote HCC migration. **A** WB was used to detected the effect of SEPT11 expression on several key factors downstream of RhoA; **B** The effect of RhoA on SEPT11-activated LIMK/cofilin pathway was measured by WB; **C** The effect of ROCK1 inhibitor Y27632 on SEPT11-activated LIMK/cofilin pathway was detected by WB; **D** The effect of Y27632 on SEPT11-regulated actin arrangement and increase was tested by cellular immunofluorescence; **E** Scratch assay was used to detect the effect of RhoA on cell migration by inhibited by KO-SEPT11; **F** Transwell assay was used to detect the effect of Y27632 on cell migration regulated by SEPT11.

Owing to the mechanism of SEPT11 regulating cytoskeleton is through activation of RhoA, we explored the effect of SEPT11 on the downstream factors of the RhoA pathway. We found that SEPT11 affected the expression of ROCK1, but not ROCK2 and IQGAP (Fig. 5A). It is known that the phosphorylation of LIMK and cofilin, the downstream factors of ROCK1, regulates the dynamic changes of actin. We further explore the effect of SEPT11 on ROCK1 and its downstream signals. The results showed that the expression of p-LIMK and p-cofilin was significantly inhibited by KO-SEPT11, but the overexpression of RhoA rescued the inhibition of ROCK1 and its downstream signal caused by KO-SEPT11 (Fig. 5B). Next, we treated SEPT11-overexpressing cells with Y27632, a specific inhibitor of ROCK1, and found that Y27632 could significantly inhibit the LIMK/cofilin pathway activated by SEPT11 overexpression (Fig. 5C). ROCK1 is known to be a direct downstream factor of RhoA, so these results suggest that SEPT11 regulates the ROCK1/LIMK/cofilin pathway through RhoA. At the same time, Y27632 significantly inhibited the increase of actin fibers, fiber structure thickening and orderly arrangement induced by overexpression of SEPT11 without affecting the expression of SEPT11 and RhoA (Fig. 5D). Scratch experiments showed that overexpression of RhoA rescued the inhibition of cell migration induced by KO-SEPT11 (Fig. 5E). Migration experiments showed that Y27632 significantly inhibited cell migration promoted by overexpression of SEPT11 (Fig. 5F). Thus, SEPT11 promotes activation of the ROCK1/LIMK/cofilin pathway, regulating cytoskeleton and migration in HCC cells.

### SEPT11 activates FAK/Src/paxillin signaling, promoting HCC cell adhesion, and migration

**Fig. 6:**
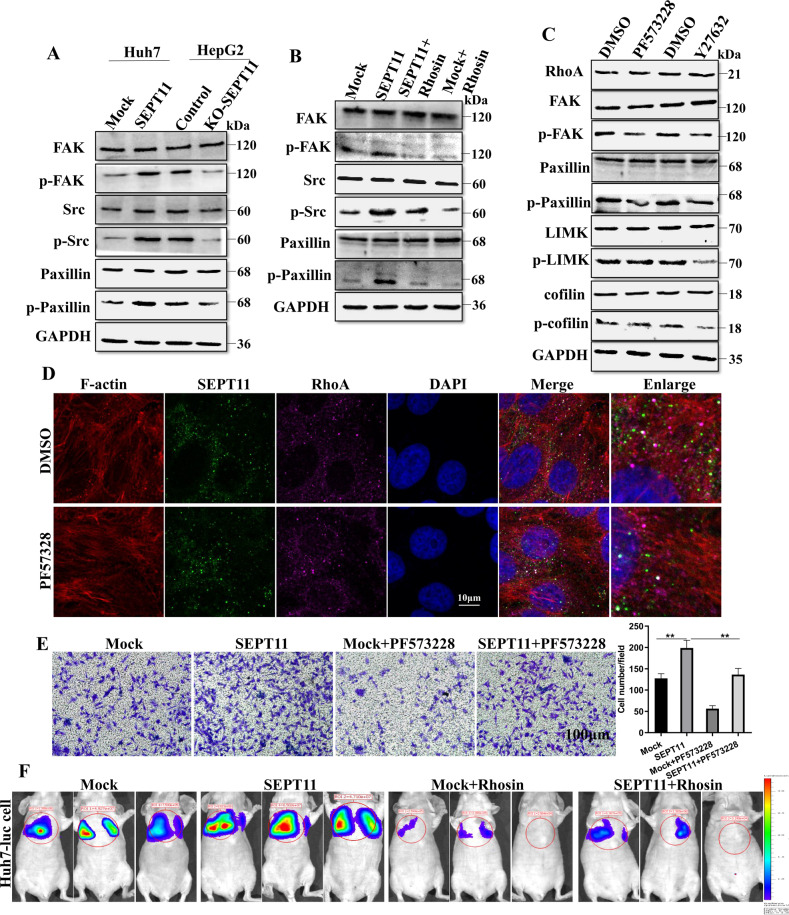
SEPT11 activates FAK/Src/paxillin signaling to promote HCC migration. **A** WB detection of the effect of SEPT11 expression on FAK/Src/paxillin signaling; **B** WB detection of the effect of RhoA on SEPT11-activated FAK/Src/paxillin signaling; **C** WB detection of Y27632 and FAK inhibitor PF573228 on FAK/Src/paxillin and LIMK/cofilin pathways; **D** The effect of PF573228 on actin arrangement and thickness detected by cellular immunofluorescence. **E** Detection of the effect of PF573228 on SEPT11-promoted cell migration; **F** Huh7-luciferase cells were injected into the tail vein of nude mice, and two groups of mice were treated with Rhosin (30 mg/kg) by intraperitoneal injection, then observed and photographed on the 28th day.

RhoA not only regulates cytoskeleton reorganization, but is closely related to FAK signaling [36, 37]. FAK is associated with the dynamic assembly of focal adhesions (FAs) and the FAK/Src/paxillin signaling cascade is known to be involved in cell migration and adhesion [29]. This study has demonstrated that SEPT11 activates RhoA to function, and GSEA analysis also showed that SEPT11 regulates FAs (Supplementary Fig. 4A). Given these available results, we explored the effect of SEPT11 on FAs. We found that overexpression of SEPT11-activated FAK/Src/paxillin pathway, but knockdown of SEPT11 inhibited this signaling pathway (Fig. 6A). These results suggest that SEPT11 can mediate the activation of the FAK/Src/paxillin pathway. What is the upstream and downstream relationships between FAK signaling and RhoA/ROCK1 signaling in SEPT11-mediated HCC cell migration? We found that Rhosin could significantly inhibit the FAK/Src/paxillin pathway and the activation of this signaling pathway by SEPT11 (Fig. 6B). Cells were treated with FAK inhibitor PF-573228 or ROCK1 inhibitor Y27632, and found that PF-573228 inhibited FAK signaling but not affect RhoA/LIMK/cofilin signaling, while Y27632 inhibited both pathways (Fig. 6C). Meanwhile, PF-573228 also did not affect the number and orderly arrangement of actin fibers (Fig. 6D). So it is likely that RhoA-ROCK1 play a role upstream of FAK/Src/ paxillin signaling to enhance HCC cell adhesion and migration. Transwell migration and invasion experiments found that PF-573228 significantly inhibited cell migration and invasion promoted by SEPT11 overexpression (Fig. 6E; Supplementary Fig. 4F). The rescue experiments in vivo revealed that Rhosin nearly abolished SEPT11-promoted HCC metastasis (Fig. 6F). Thus, SEPT11 targets RhoA, thereby regulating cytoskeleton rearrangement and abnormal cell adhesion through ROCK1/cofilin and FAK/paxillin signaling pathways, promoting invasion and migration of HCC.

### SEPT11 activates RhoA by promoting the binding of GEF-H1 to RhoA

**Fig. 7:**
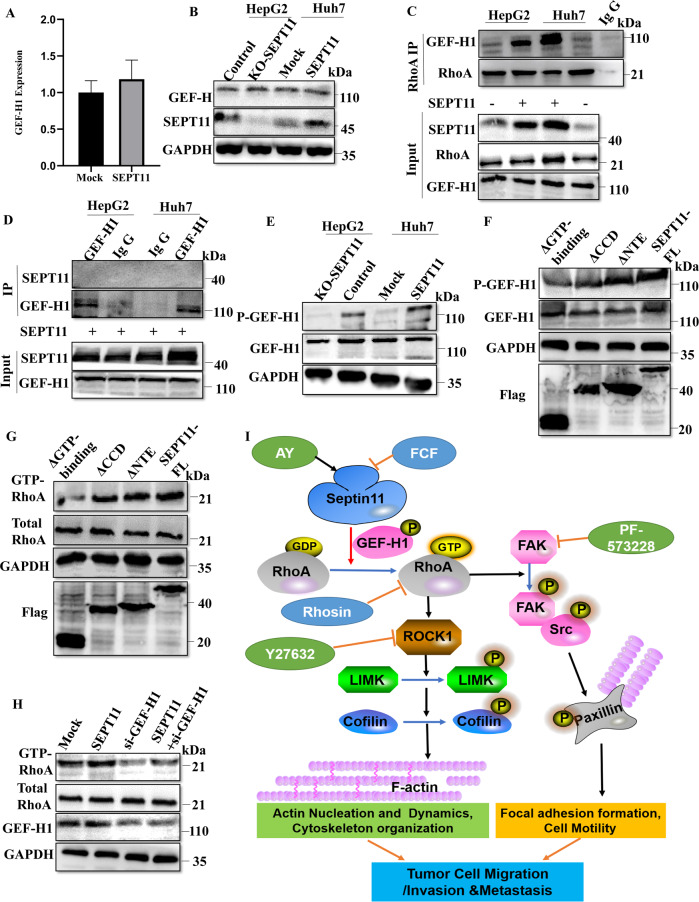
SEPT11 regulates the activation of RhoA through GEF-H1. **A**, **B** QPCR and WB were used to detect the transcriptional expression and protein content of GEF-H1; **C** CO-IP and WB were used to detect the effect of SEPT11 overexpression on GEF-H1 and RhoA binding; **D** CO-IP and WB were used to detect the binding of SEPT11 and GEF-H1 in HepG2 and Huh7 cells of SEPT11-overexpressed; **E** WB was used to detect the influence of SEPT11 on phosphorylation of GEF-H1 Ser886; **F** WB was used to detect the effect of each structural domain of SEPT11 on Phospho-GEF-H1; **G** The activation of RhoA promoted by SEPT11 was abolished after removal of the GTP-binding domain; **H** GEF-H1 can influence the activation of RhoA by SEPT11; **I** The diagram shows that SEPT11 promotes the activation of RhoA through GEF-H1, then RhoA regulates cytoskeletal organization and FA dynamics by activating LIMK/cofilin and FAK/Src pathways, thereby promoting the invasion and migration of HCC.

It is well known that Rho GTPases are activated by guanine nucleotide exchange factors (GEFs), and multiple guanine nucleotide exchange factors have been shown to activate Rho GTPase, including GEF-H1, ARHGEF3, NET1 and so on [38]. GEF-H1 was of focus, because it was considered as RhoA-specific GEF and its catalytic activity toward RhoA is changed through microtubule dynamics influenced by Septins [39–41]. Therefore, we explored that whether SEPT11 affects the activity of RhoA through GEF-H1. Firstly, we explored whether SEPT11 affects the expression of GEF-H1. We found that SEPT11 did not significantly affect the transcriptional expression and protein content of GEF-H1(Fig. 7A, B). However, we found SEPT11 can promote the binding of GEF-H1 to RhoA (Fig. 7C). It is well known that GEF-H1 binds to RhoA can increase the GTP-RhoA form and activate RhoA [42]. Thus, SEPT11 may affects RhoA activity through GEF-H1. Co-IP was used to detect the combination between SEPT11 and GEF-H1, but we did not find the interaction between them (Fig. 7D). Phosphorylation of GEF-H1 Ser886 can promote the binding of GEF-H1 to RhoA, and activate RhoA [43]. We found that SEPT11 can promote the level of Phospho-GEF-H1 Ser886 (Fig. 7E). We further explored the effect of each structural domain of SEPT11 on Phospho-GEF-H1. Then we found that the phosphorylation effect of GEF-H1 Ser886 promoted by SEPT11 was abolished after removal of the GTP-binding domain (Fig. 7F; Supplementary Fig. 4D). GTP-binding domain of SEPT11 is also critical for activation of RhoA (Fig. 7G). Further, we knocked down GEF-H1 though siRNA, which inhibited the activation of RhoA by SEPT11 (Fig. 7H). Furthermore, in the ΔGTP-binding domain of SEPT11 fragment treatment groups, the GEF-H1 binding to RhoA less than the other SEPT11 fragment treatment groups (Supplementary Fig. 4E). Therefore, SEPT11 facilitates the binding of GEF-H1 to RhoA by promoting Phospho-GEF-H1 Ser886, which enhances the activity of RhoA, and the GTP-binding domain is crucial to play this role.

## Discussion

Central to the poor prognosis of HCC is regional spread and metastasis [1, 2]. Septins are an important part of the cytoskeleton, involved in cell invasion and migration, and regulate tumor development and progression [44, 45]. It has been reported that SEPT9 promotes the upregulation of MMPs expression near FAs and secretion of MMPs, thereby enhancing the migration and invasion of breast cancer cells [46]. Septin 7 plays a downstream effect in ERK3-induced migration of cancer cells, and its deletion abolishes the ability of ERK3 to promote lung cancer cell migration and invasion [47]. CDK2 interacts with SEPT2 to stabilize SEPT2 in HCC cells, and HCC with high expression of both CDK2 and SEPT2 is more prone to recurrence and may be more aggressive [48]. As a new member of the septin family, we reported the function of SEPT11 in HCC progression for the first time. In this study, we demonstrated the function of SEPT11 in promoting the metastasis of HCC, and preliminarily explored the related molecular mechanism of its promoting HCC metastasis.

Our previously results found that high expression of LncRNA AY is closely associated with poor prognosis and metastasis of patients with HCC [5]. In the process of exploring the function of AY in promoting HCC, we accidentally discovered a tumor-promoting gene SEPT11. We found that the expression of SEPT11 in HCC is regulated by AY. SEPT11 is an important target gene of AY to promote HCC progression. SEPT11 is widely expressed in various human tissues. We found that SEPT11 upregulation in HCC was significantly correlated with tumor differentiation, vascular invasion and predicted a poor prognosis. Furthermore, SEPT11 promoted invasion and migration in HCC cells in vitro and tumor metastasis in vivo, which is consistent with the functions of other septins in tumors. It has been reported that the elevated expression of SEPT9 and SEPT2 in glioma tissues and cell lines not only promote cell invasion but also enhance cell proliferation [49]. Overexpression of SEPT6 significantly promoted the proliferation, cell cycle transition, migration and invasion of HCC cells, and SEPT6 gene knockout has a significant inhibitory effect on the survival of HCC cell lines [50].

However, our study found that SEPT11 had no significant effect on tumor cell proliferation either in vivo or in vitro, and the high expression or knockout of SEPT11 also had little effect on cell proliferation-related factors. The result is different from other reported septins to a certain extent, which suggests that there are some differences in the function of diverse septins, but these results also confirm the ability of septins to regulate cell migration and invasion.

Given that migratory capability in SEPT11 has been found in vitro and in vivo, the mechanism of SEPT11 in HCC cells had important clinical values. As a cytoskeleton GTPase, the classical function of septins is to regulate the reorganization of cytoskeleton, thereby regulating cell motility [9, 10]. Actin (microfilaments), the main component of cytoskeleton, which abnormal rearrangement and polymerization were important to the migration of cancer cells. Our experiments demonstrate that SEPT11 can indeed regulate actin dynamics in HCC cells. Further GSEA analysis found that most of the signaling pathways regulated by SEPT11 were related to cell migration and invasion, and the core effectors in multiple pathways included Rho proteins. RNA-seq and ATAC-seq suggested that AY-regulated genes were enrichment in Rho GTPase activity and regulation of actin cytoskeletal. SEPT11 acts as an important downstream factor for the function of AY, which also implies that SEPT11 may be related to Rho GTPase signaling. Meanwhile, several studies have demonstrated that septins induce abnormal migration of cancer cells by regulating actin cytoskeleton signaling through Rho GTPase [29, 30, 32, 33]. Furthermore, our results are similar to those previously reports that SEPT11 indeed regulates the dynamic changes of actin by promoting the activation of RhoA, and play a role in promoting migration in HCC.

Increased migration ability was always related to high cytoskeletal tension, ordered arrangement of actin and abnormal cell adhesion [26]. RhoA and its downstream effectors are key regulators of adhesion and actin organization [35]. The study explored the effect of SEPT11 on downstream factors of RhoA. ROCK1 is the main target of RhoA. Through combining with LIM domains ROCK1 activates LIMK and its direct downstream effector cofilin, drives the turnover and spatial reorganization of F-actin [51]. Actin reorganization was reregulated by cofilin, as actin-depolymerizing factor of actin filaments, the phosphorylation level of cofilin regulates the cytoskeleton dynamics [26]. Prior work has shown that ROCK/LIMK/cofilin axis is important to promote migration of cancer [52]. In this study, we found that SEPT11 regulates the ROCK1/LIMK/cofilin pathway through RhoA, modulates actin cytoskeletal reorganization through phosphorylating and inactivating cofilin, and ultimately induces cell migration. The ROCK1-specific inhibitor Y27632 significantly suppressed the ROCK1/LIMK/cofilin pathway activated by SEPT11, inhibited polymerization of F-actin, decreased cell migration ability. These results were consistent with previous findings that LIMK and cofilin were downstream effectors of RhoA/ROCK pathway to regulate actin organization.

Cell adhesion and migration involve FA remodeling that is related to cytoskeletal organization and associated intracellular signals [53]. Cell adhesion is mediated by two core effectors FAK and Src within cells [54]. FAK is a protein tyrosine kinase that is activated by Rho protein, integrin and other signals, thus performing a variety of functions, such as attachment, migration, invasion and so on [55]. FAK is also a scaffolding protein that binds to Src, then can form FAK-Src complex and regulate downstream FA proteins such as paxillin. After phosphorylation of Paxlin Y118 regulated by FAK-Src complex, other adhesion molecules were recruited to mediate cell adhesion [56]. Our bioinformatics analysis showed that SEPT11 regulates the focal adhesion pathway. SEPT11 activates FAK/Src/paxillin pathway and thus promotes tumor migration, but both Rhosin and Y27632 can significantly inhibit the activation of this signal pathway by SEPT11. Meanwhile, Rhosin also nearly abolished SEPT11-promoted HCC metastasis. FAK signaling is closely related to Rho GTPase, RhoA is critical for modulating the actin-associated adhesion during cell migration [57]. Cell treatment with inhibitors of contractility significantly reduces cytoskeletal tension, then suppress FAK signaling [58]. FA dynamics and cytoskeletal organization make concerted efforts to drive cell migration [59]. Our work links SEPT11, F-actin and FAs to tumor cell motility, migration and metastasis for the first time.

SEPT11 did not form a firm complex with RhoA to influence its activity. Thus, “How SEPT11 affects RhoA activity”. GEFs specifically catalyze the exchange of GDP for GTP, which activates Rho GTPases. GEF-H1 is considered as RhoA-specific GEF, and can stimulate guanine nucleotide exchange of RhoA but is weak or inactive toward RAC, Cdc42, TC10 or Ras [60, 61]. The catalytic activity of GEF-H1 toward RhoA is changed through microtubule dynamics [39, 40], while septins (including SEPT11) associate with microtubule bundles and promote as well as guide microtubule growth in living cells, to influence their dynamics [62, 63]. Therefore, we hypothesized that SEPT11 affects the activity of RhoA through GEF-H1. We found that SEPT11 did not significantly affect the transcriptional expression and protein content of GEF-H1, but it could promote the phosphorylation of GEF-H1 Ser886 and the binding of GEF-H1 to RhoA. Further, the knocked down of GEF-H1 can inhibite the activation of RhoA by SEPT11. Phosphorylation of GEF-H1 Ser886 is important for GEF-H1-driven activation of RhoA [43]. Thus, SEPT11 affects RhoA activity through GEF-H1. We further found that GTP-binding domain of SEPT11 was critical for phosphorylation of GEF-H1 Ser886 and activation of RhoA. In sum, our study showed that SEPT11 affects RhoA activity through EGF-H1, and the GTP-binding domain is crucial to play this role.

In conclusion, SEPT11 is an important downstream factor of AY in promoting HCC. SEPT11 promotes the activation of RhoA through GEF-H1, then RhoA regulates cytoskeletal organization and FA dynamics by activating LIMK/cofilin and FAK/Src pathways, thereby promoting the invasion and migration of HCC (Fig. 7I). These data provide evidence for the involvement of SEPT11 in tumor progression and suggest that SEPT11 may serve as a novel prognostic marker and therapeutic target for HCC.

## Supplementary information

aj-checklist-CDDIS-22-2493R

Table 1

Table 2

Supplementary Fig S1

FigureS2

FigureS3

FigureS4

Supplementary Figure legends

Full and uncropped western blots

Cell STR Authentication

Cell STR Authentication

293T STR

Cell STR Authentication

## Data Availability

The data needed to evaluate the conclusions in the paper are present in the paper and/or the supplementary materials, including full and uncropped western blots. Raw datasets generated and/or analyzed of this study are available from the corresponding author, upon reasonable request.
