# Supplementary material for: Septin11 promotes hepatocellular carcinoma cell motility by activating RhoA to regulate cytoskeleton and cell adhesion
Source: Cell Death Dis. 2023 Apr 20;14(4):280. doi: 10.1038/s41419-023-05726-y (PMC10119145; doi:10.1038/s41419-023-05726-y)
Supplement: Supplementary file 2 — Table 1 [file 41419_2023_5726_MOESM2_ESM.docx]

**Supplementary Table 1 Patient characteristics**

| ***Clinicopathological data*** |  | **Frequency** | **Percentage** | |
| --- | --- | --- | --- | --- |
| ***Age (years)*** |  |  |  | |
|  | mean (SD) | 51.3 (±11.14) | |  |
|  | range | 19-76 |  | |
| ***Gender*** |  |  |  | |
|  | male | 65 | 85.5% | |
|  | female | 11 | 14.5% | |
| ***HBV*** |  |  |  | |
|  | negative | 7 | 9.2% | |
|  | positive | 67 | 88.2% | |
|  | unknown | 2 | 2.6% | |
| ***HCV*** |  |  |  | |
|  | negative | 7 | 9.2% | |
|  | positive | 63 | 82.9% | |
|  | unknown | 6 | 7.9% | |
| ***AFP(α-fetoprotein)*** |  |  |  | |
|  | <200 μg/L | 34 | 44.7% | |
|  | ≥200 μg/L | 39 | 51.3% | |
|  | unknown | 3 | 4.0% | |
| ***Tumor size*** |  |  |  | |
|  | <5cm | 35 | 46.1% | |
|  | ≥5cm | 41 | 53.9% | |
| ***Tumor multiplicity*** |  |  |  | |
|  | single | 67 | 88.2% | |
|  | multiple | 9 | 11.8% | |
| ***Microvascular invasion*** |  |  |  | |
|  | negative | 47 | 61.8% | |
|  | positive | 25 | 32.9% | |
|  | unknown | 4 | 5.3% | |
| ***Tumor capsule*** |  |  |  | |
|  | well differentiated | 33 | 43.4% | |
|  | Poorly differentiated | 42 | 55.3% | |
|  | unknown | 1 | 1.3% | |
| ***Edmondson grade*** |  |  |  | |
|  | low (Grade 1/2) | 7 | 9.2% | |
|  | high (Grade 3/4) | 65 | 85.5% | |
|  | unknown | 4 | 5.3% | |
