# Supplementary material for: Septin11 promotes hepatocellular carcinoma cell motility by activating RhoA to regulate cytoskeleton and cell adhesion
Source: Cell Death Dis. 2023 Apr 20;14(4):280. doi: 10.1038/s41419-023-05726-y (PMC10119145; doi:10.1038/s41419-023-05726-y)
Supplement: Supplementary file 3 — Table 2 [file 41419_2023_5726_MOESM3_ESM.docx]

**Supplementary Table 2 Correlation between expression of SEPT11 and clinicopathological parameters in HCC patients (n = 76)**

| **Variables** | **Low SEPT11** | | **High SEPT11** | | ***P*** |
| --- | --- | --- | --- | --- | --- |
|  | No. of | % | No. of | % |  |
|  | patients |  | patients |  |  |
|  | (n=17) |  | (n=59) |  |  |
| Age (years) |  |  |  |  | 0.83 |
| ≤46 | 7 | 41.2% | 26 | 44.1% |  |
| >46 | 10 | 58.8% | 33 | 55.9% |  |
| Tumor size (cm) |  |  |  |  | 0.23 |
| <5cm | 10 | 70.6% | 25 | 39.0% |  |
| ≥5cm | 7 | 29.4% | 34 | 61.0% |  |
| Microvascular invasion |  |  |  |  | 0.017 |
| negative | 14 | 82.4% | 33 | 55.9% |  |
| positive | 1 | 5.88% | 24 | 40.7% |  |
| unknown | 2 | 11.8% | 2 | 3.39% |  |
| Edmondson grade |  |  |  |  | 0.0045 |
| low (Grade 1/2) | 5 | 29.4% | 2 | 3.39% |  |
| high (Grade 3/4) | 11 | 64.7% | 54 | 91.5% |  |
| unknown | 1 | 5.88% | 3 | 5.10% |  |
| AFP(α-fetoprotein) |  |  |  |  | 0.034 |
| <200 μg/L | 12 | 70.6% | 22 | 37.3% |  |
| ≥200 μg/L | 4 | 23.5% | 35 | 59.3% |  |
| unknown | 1 | 5.88% | 2 | 3.39% |  |
