# Supplementary figures and images for: Septin11 promotes hepatocellular carcinoma cell motility by activating RhoA to regulate cytoskeleton and cell adhesion
Source: Cell Death Dis. 2023 Apr 20;14(4):280. doi: 10.1038/s41419-023-05726-y (PMC10119145; doi:10.1038/s41419-023-05726-y)

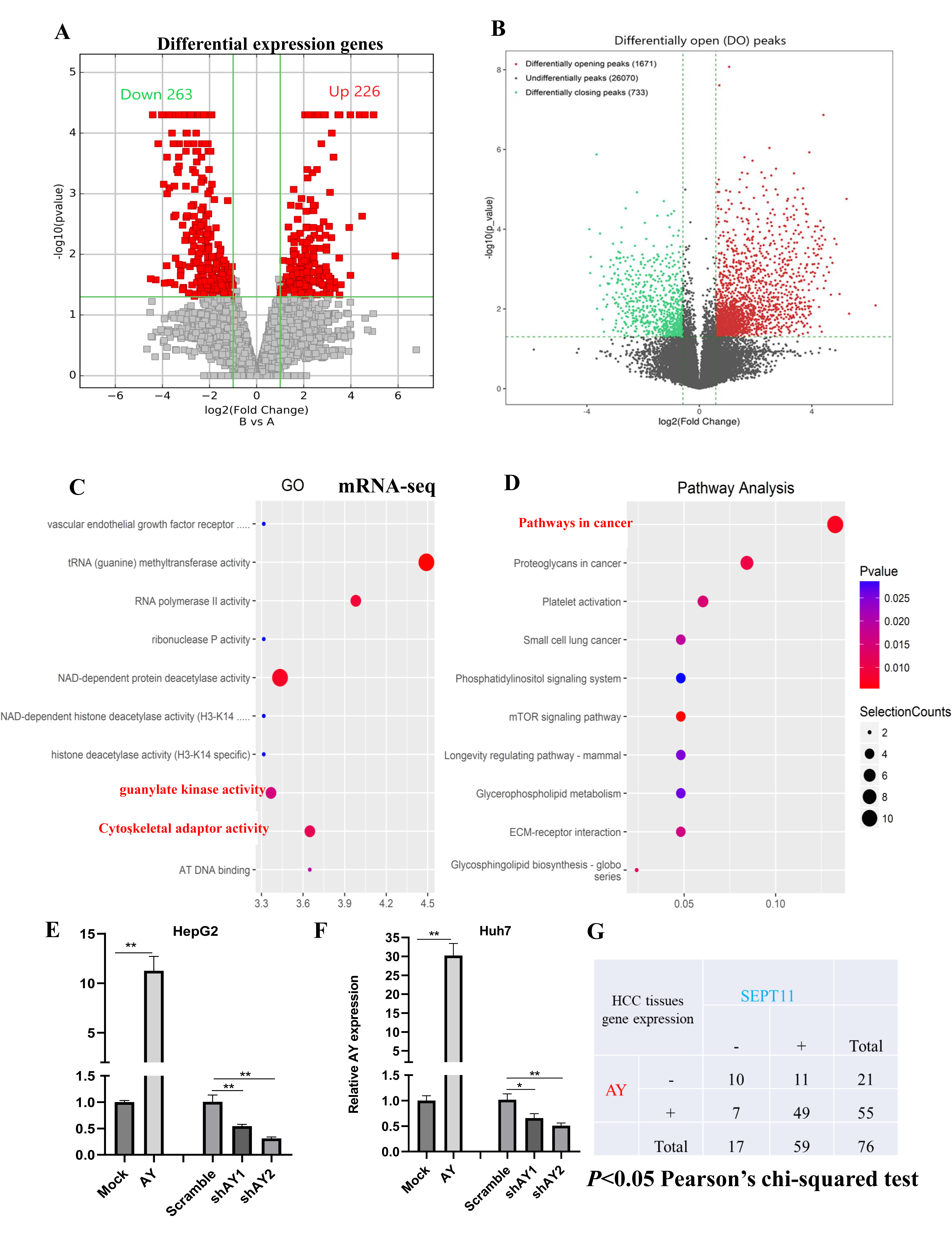

Supplement: Supplementary file 4 — Supplementary Fig S1 [file 41419_2023_5726_MOESM4_ESM.tif]

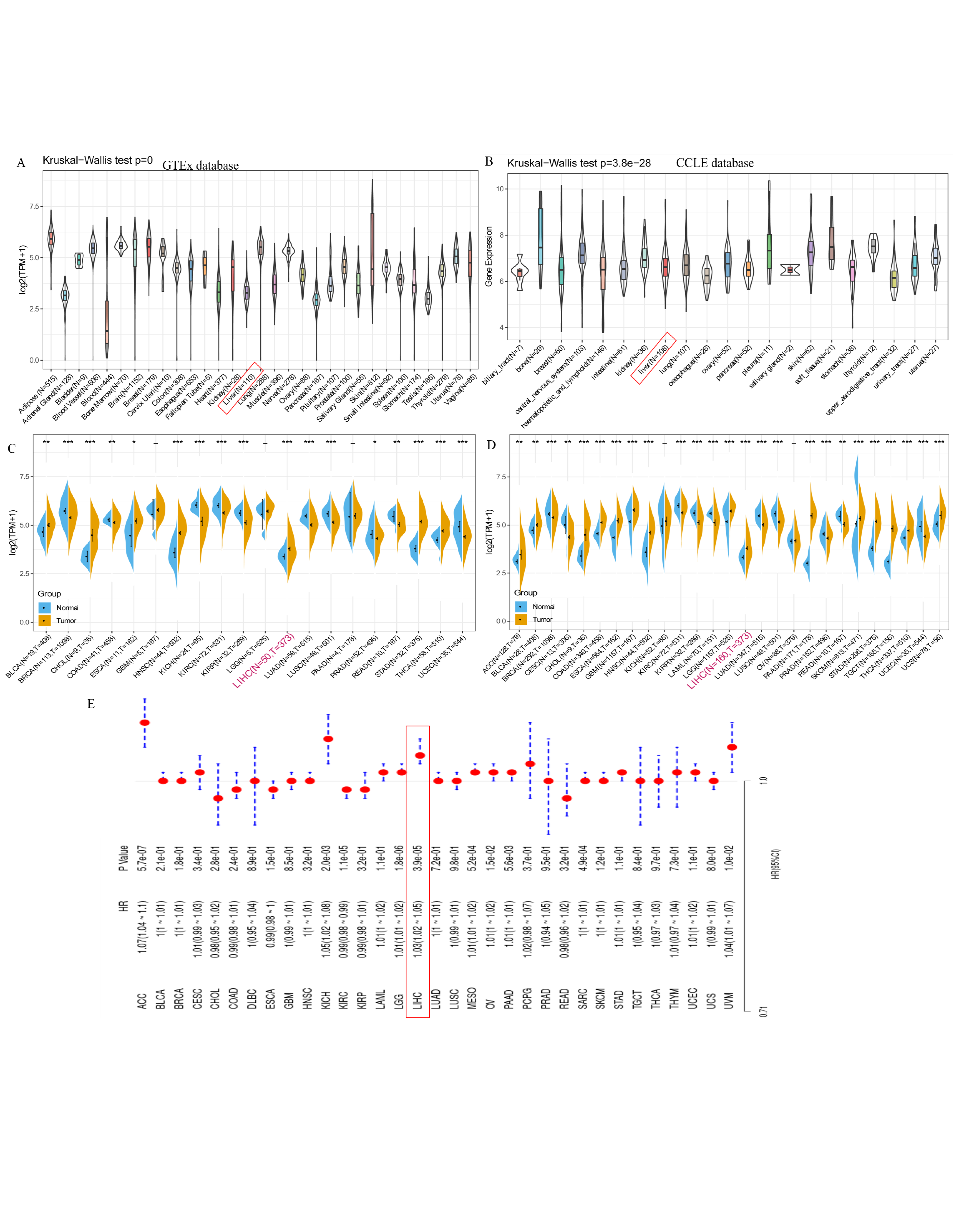

Supplement: Supplementary file 5 — FigureS2 [file 41419_2023_5726_MOESM5_ESM.tif]

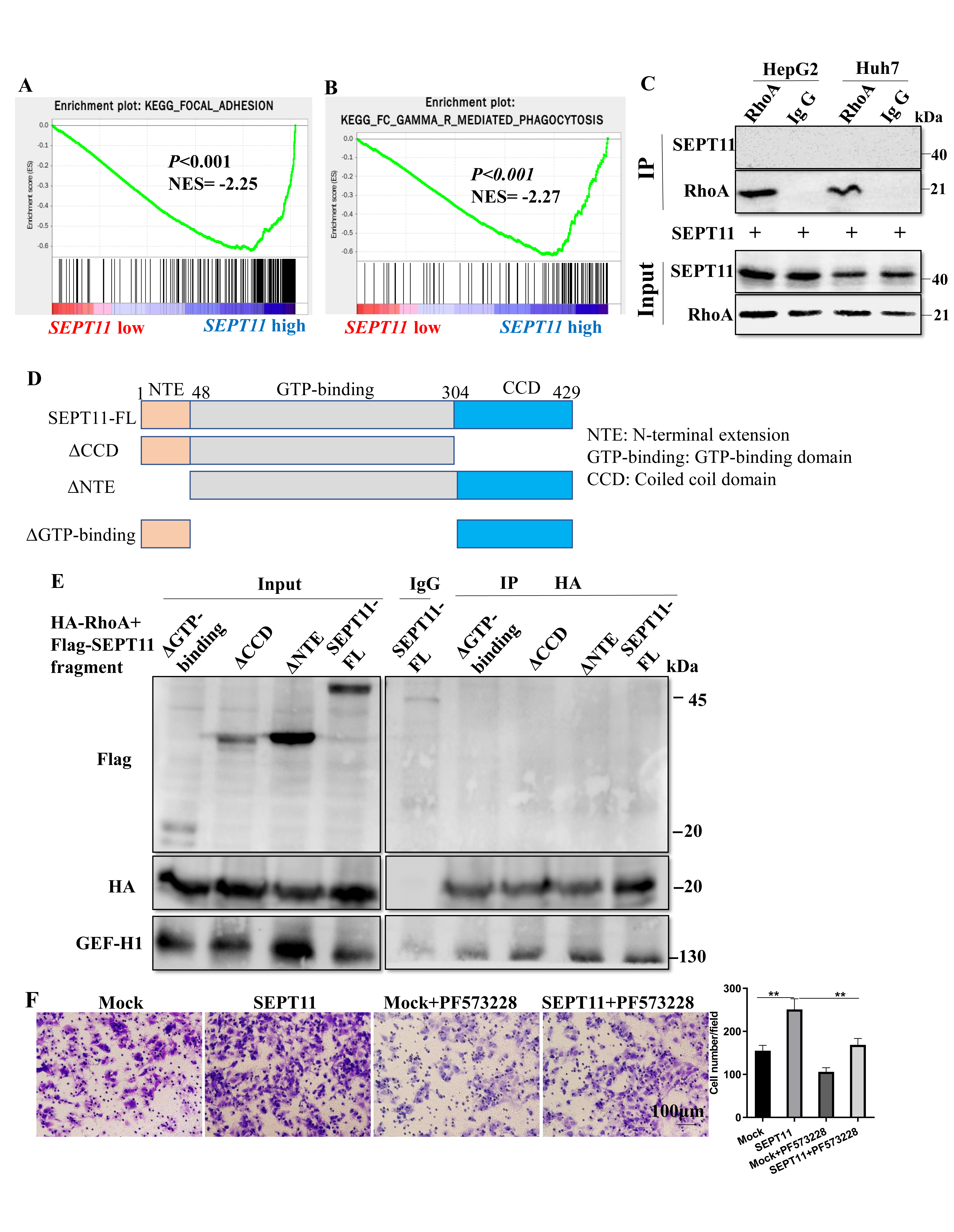

Supplement: Supplementary file 7 — FigureS4 [file 41419_2023_5726_MOESM7_ESM.tif]

**Fig 1**

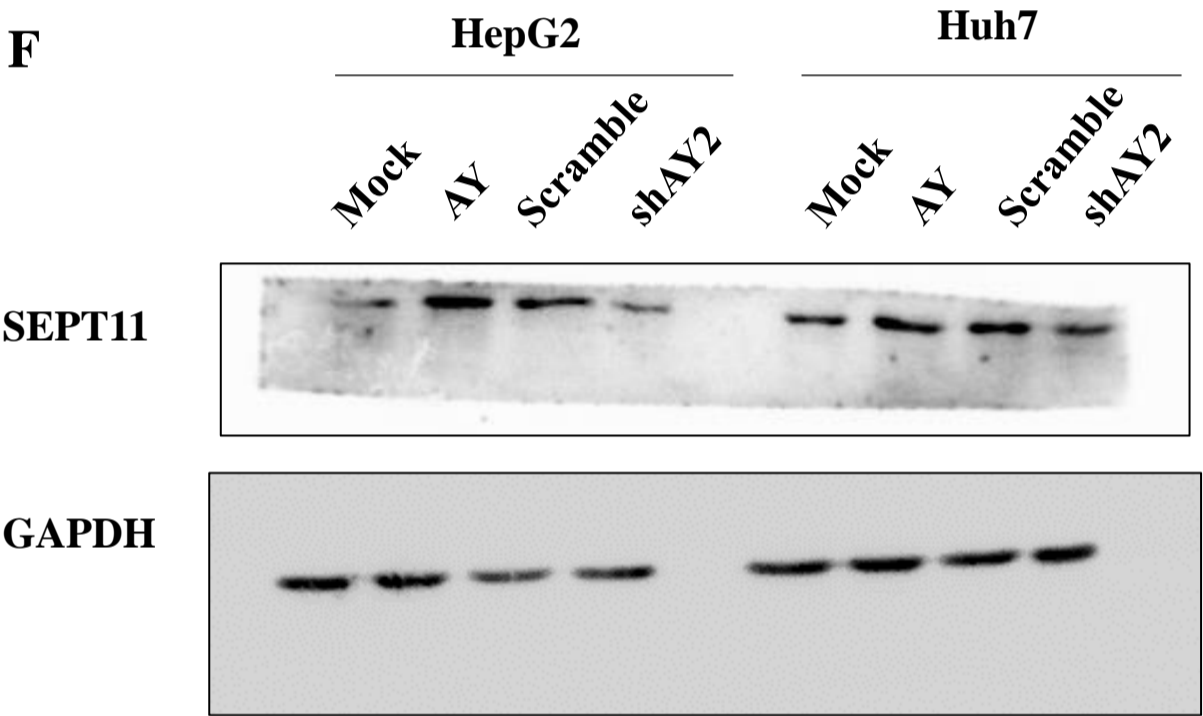

**Fig 2**

**G**

**SEPT11**

**GAPDH**

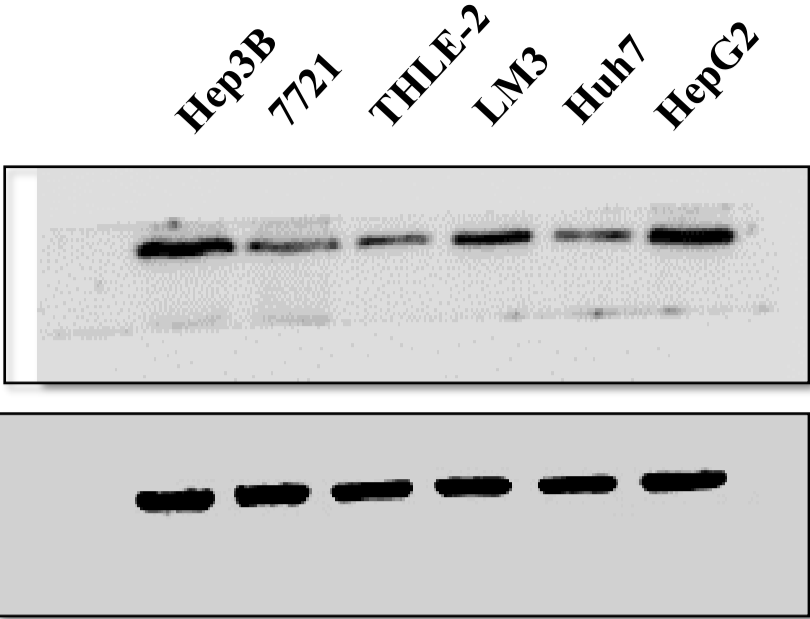

**H**

**SEPT11**

**GAPDH**

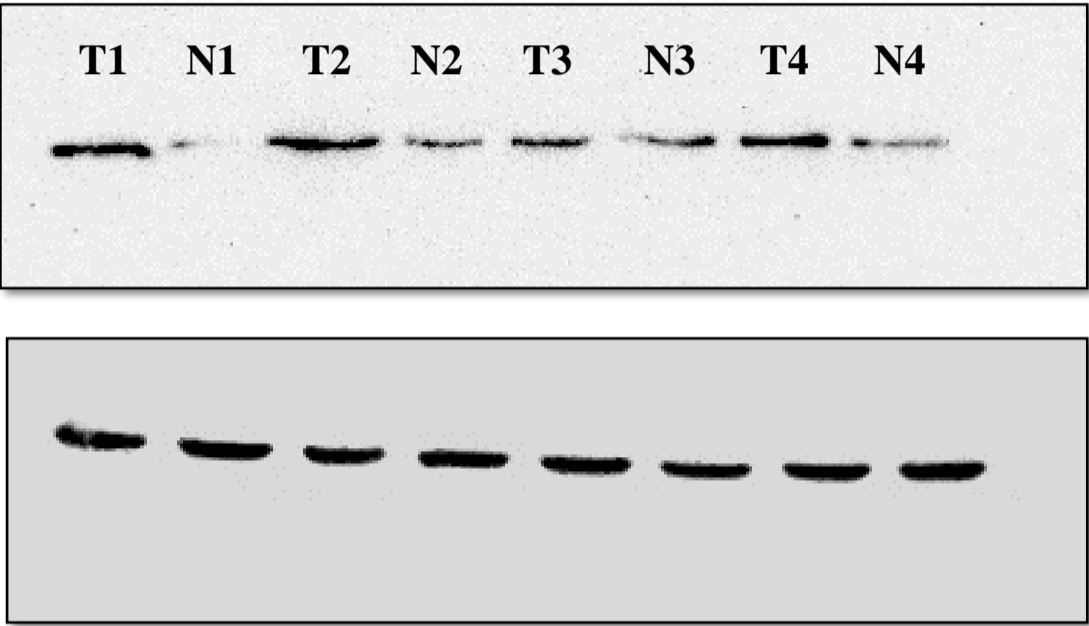

**SEPT11**

**GAPDH**

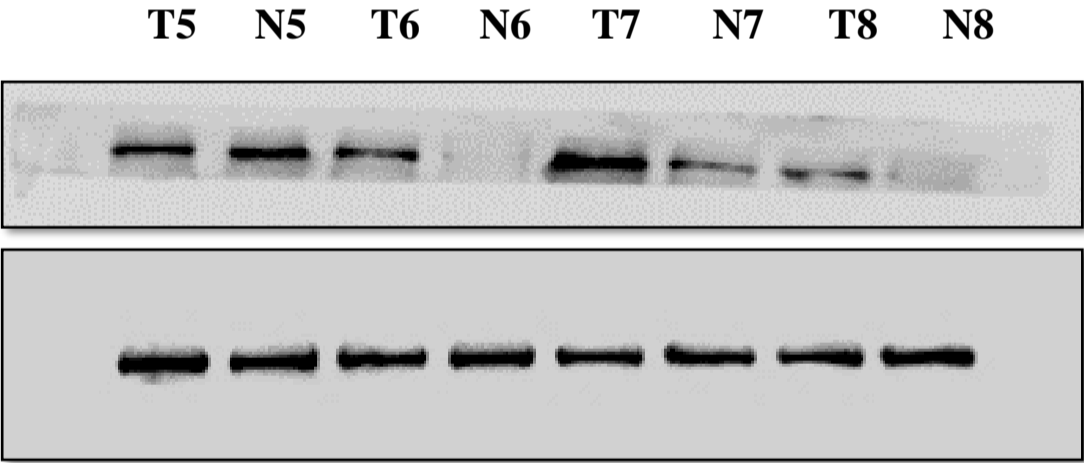

**SEPT11**

**GAPDH**

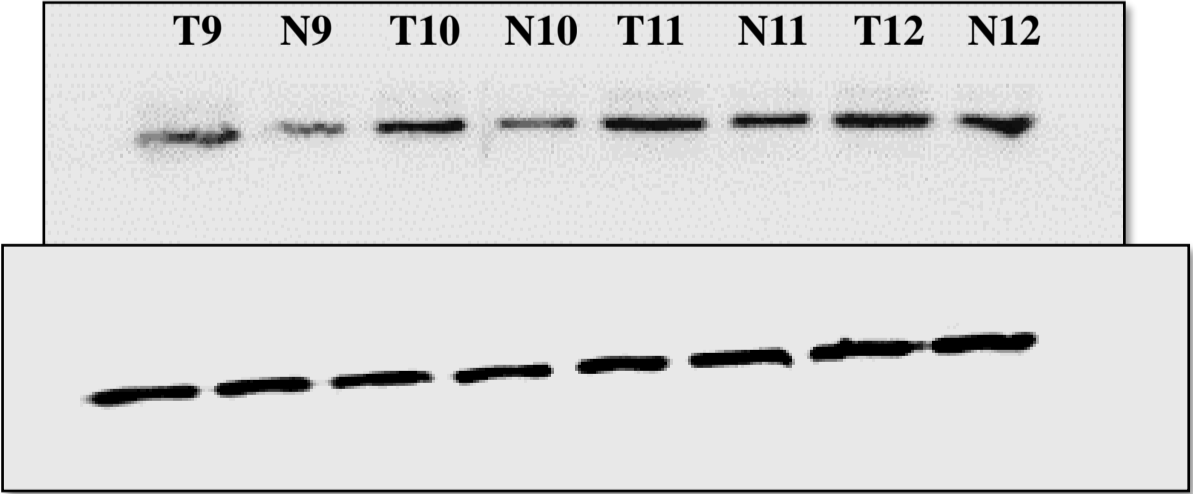

Fig3

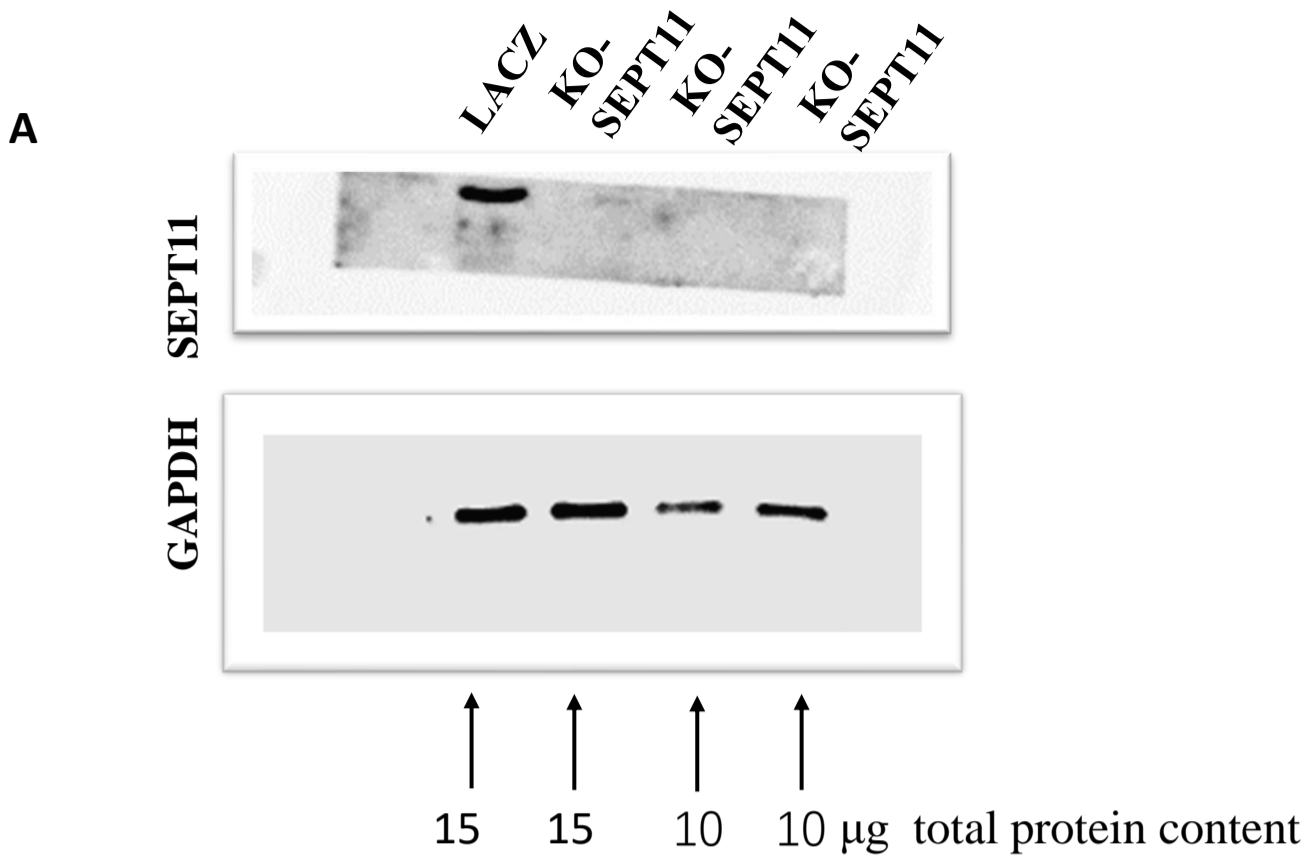

Fig S3

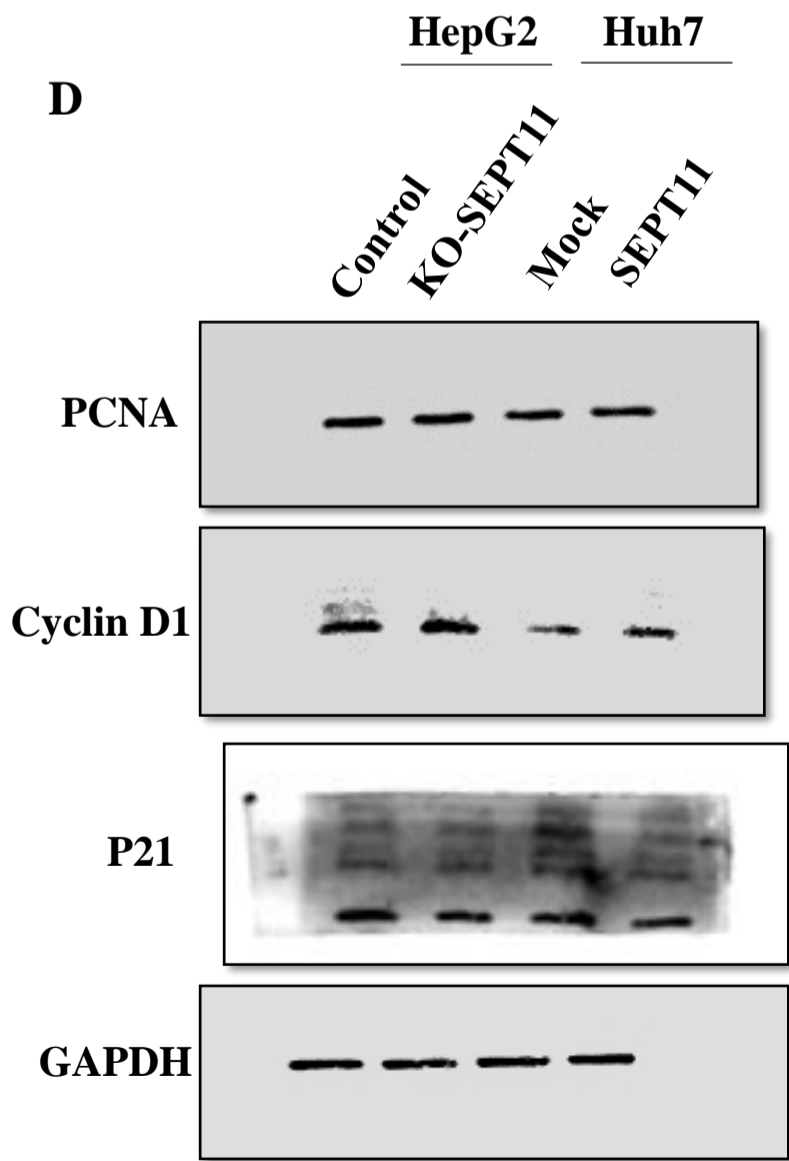

Fig S4

C

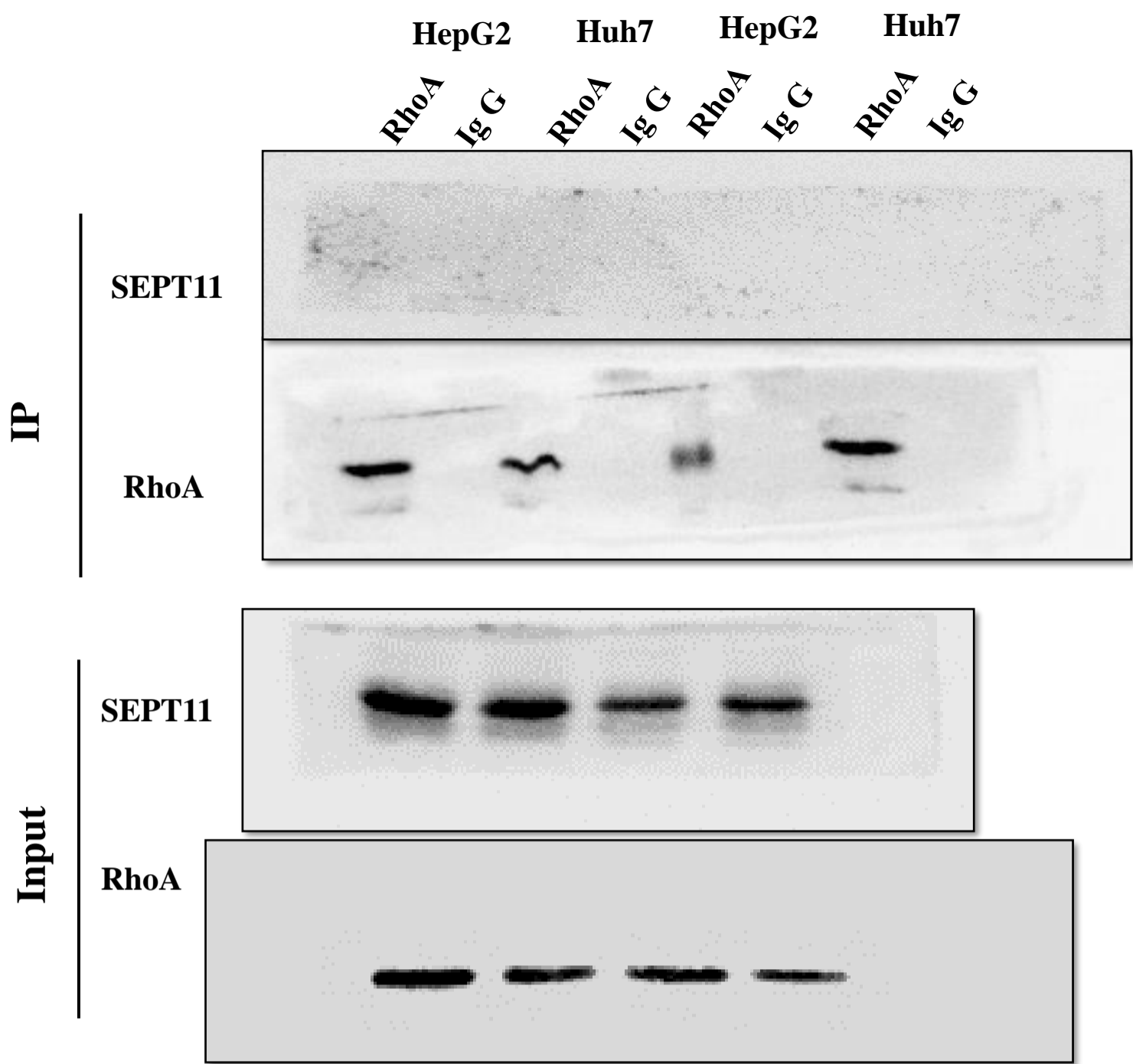

E

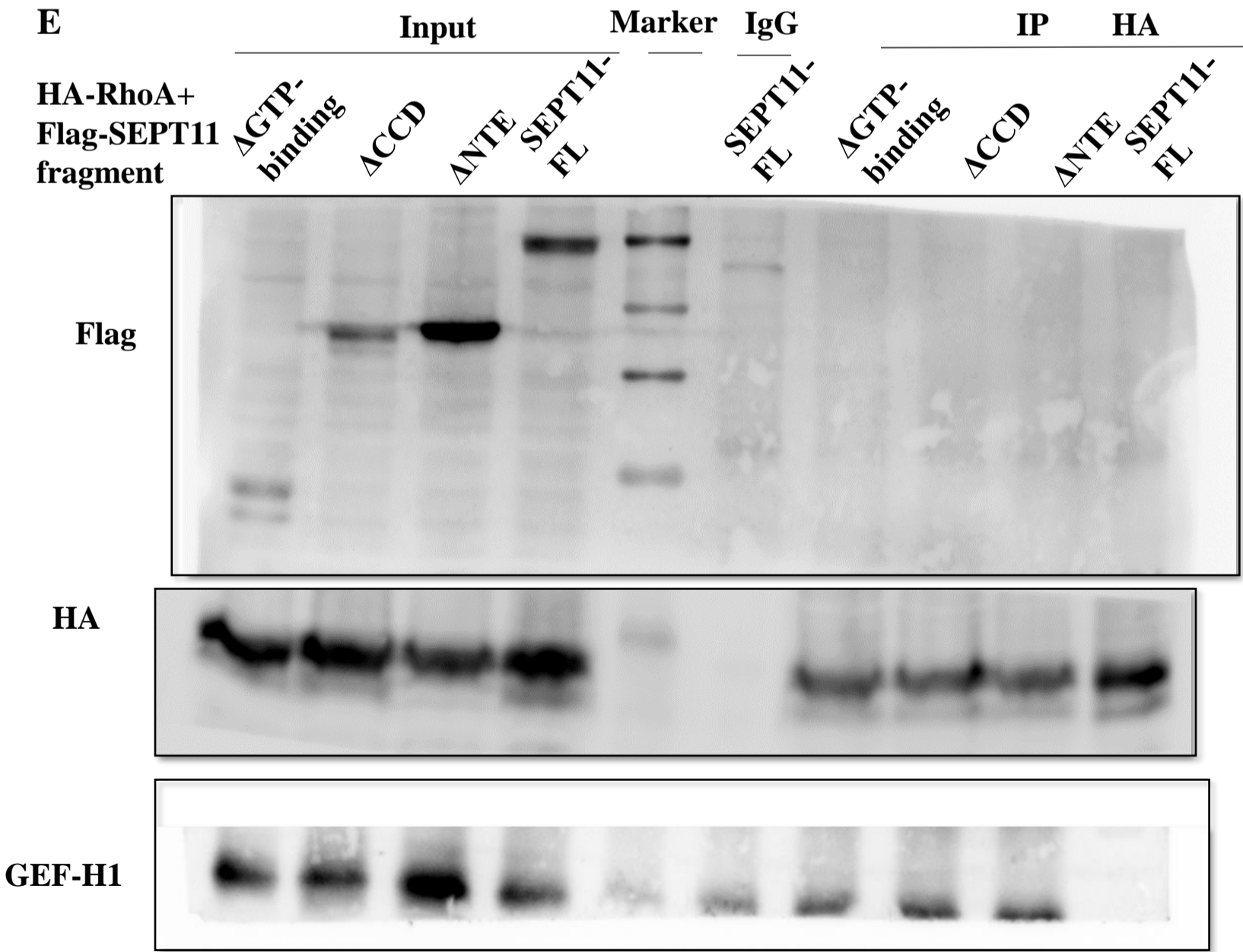

Fig4

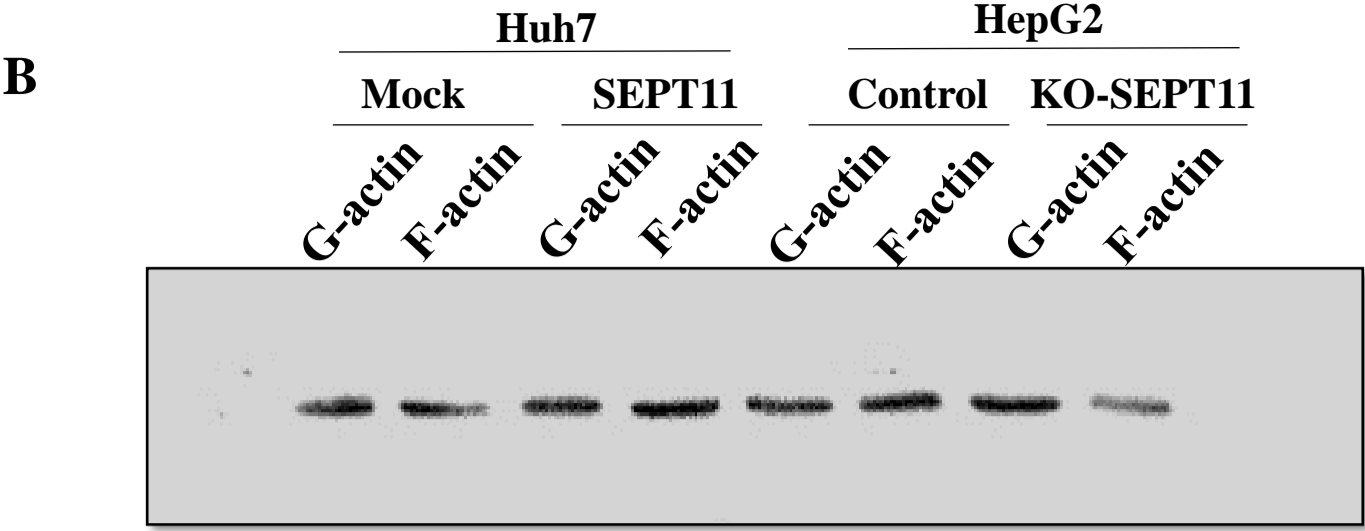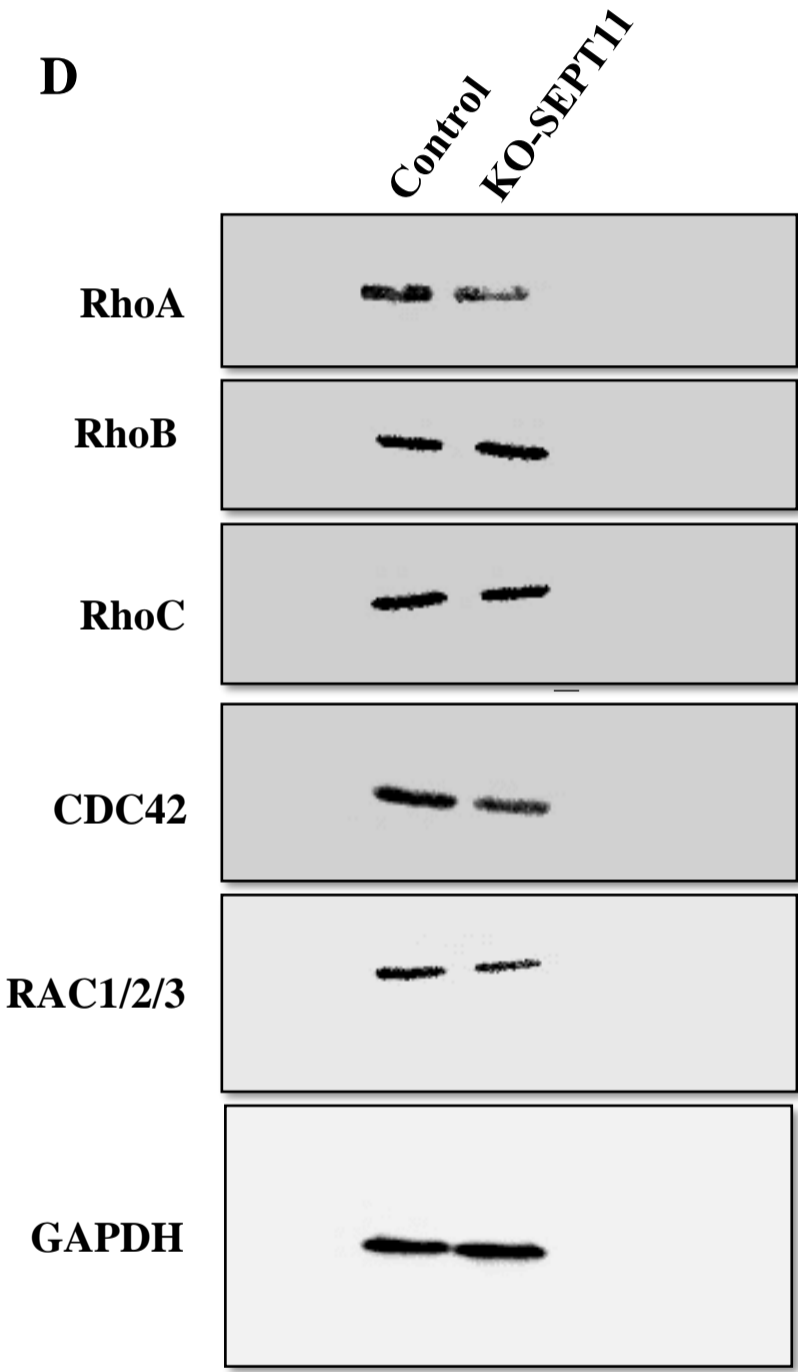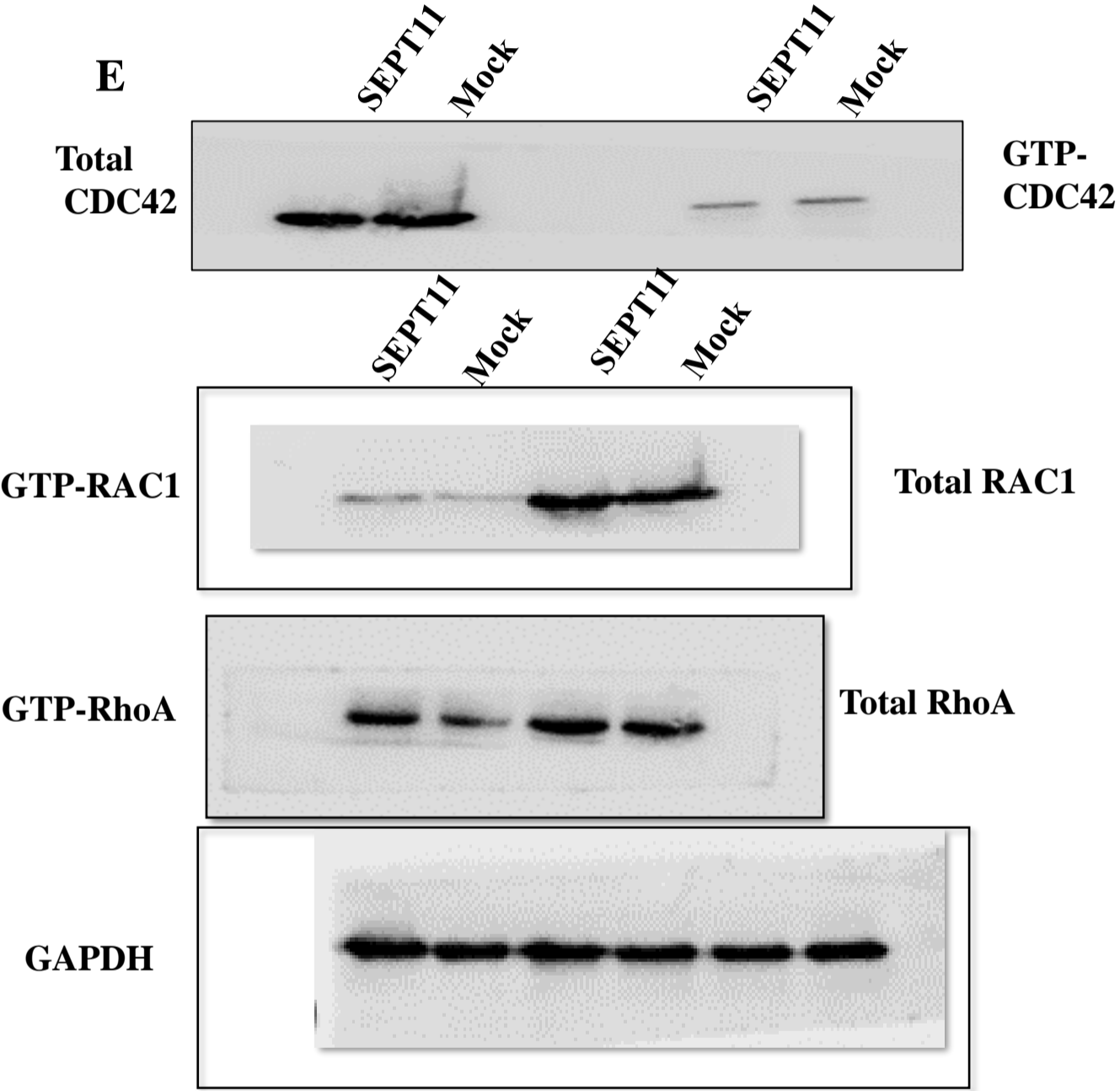

Fig4

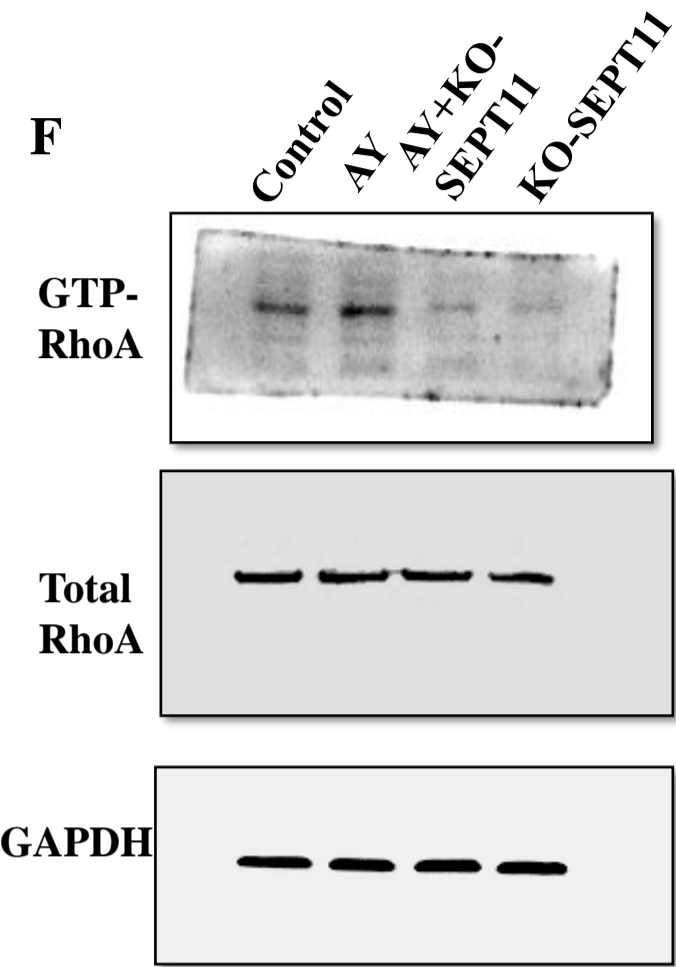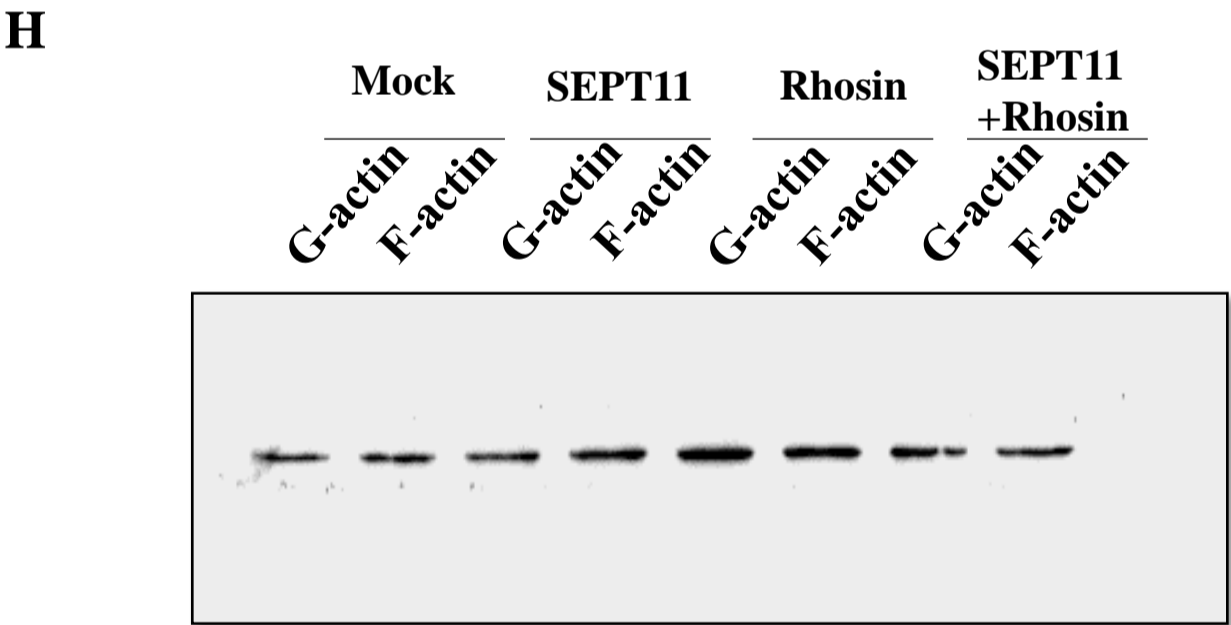

**Fig5**

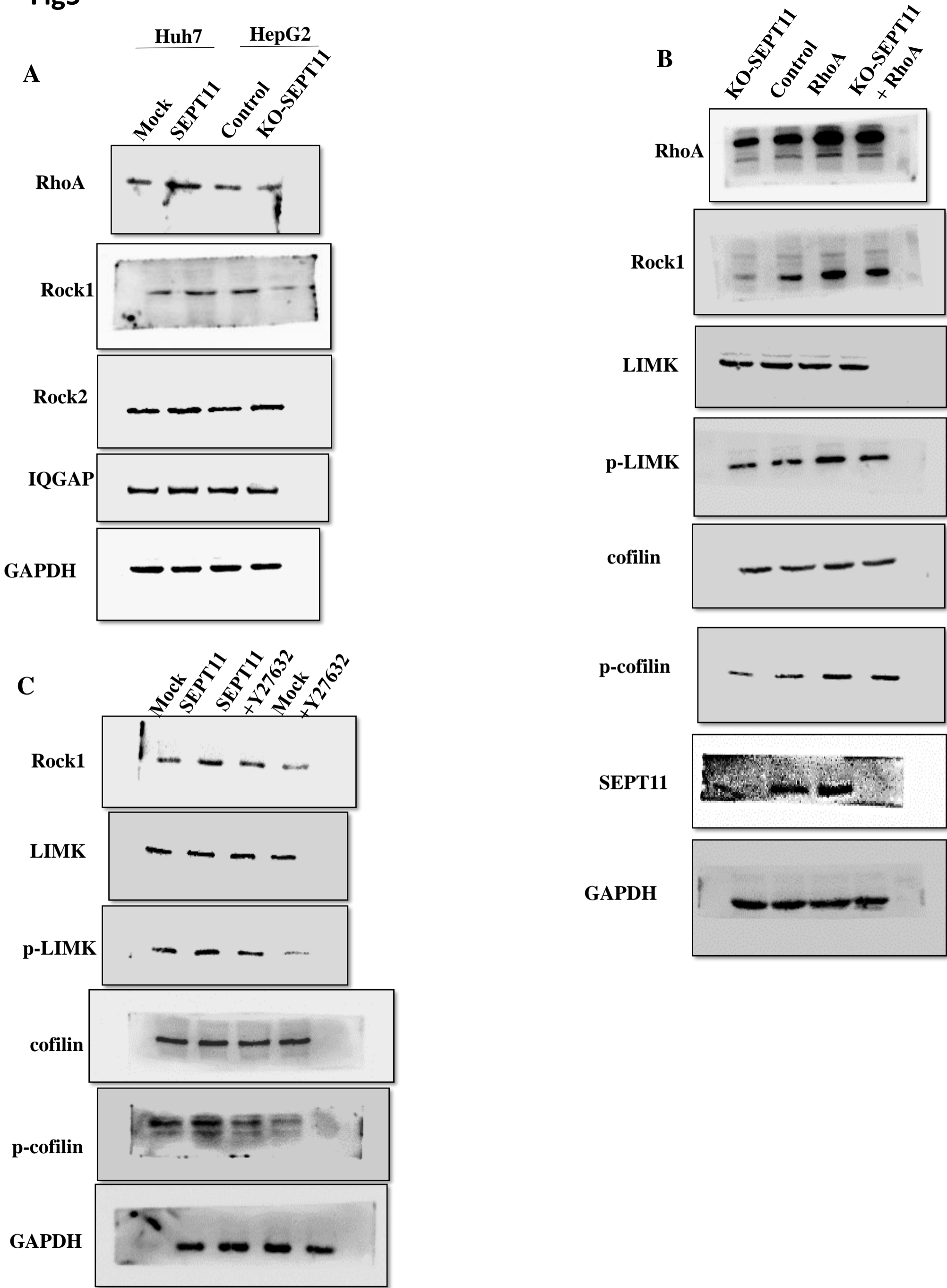

Fig6

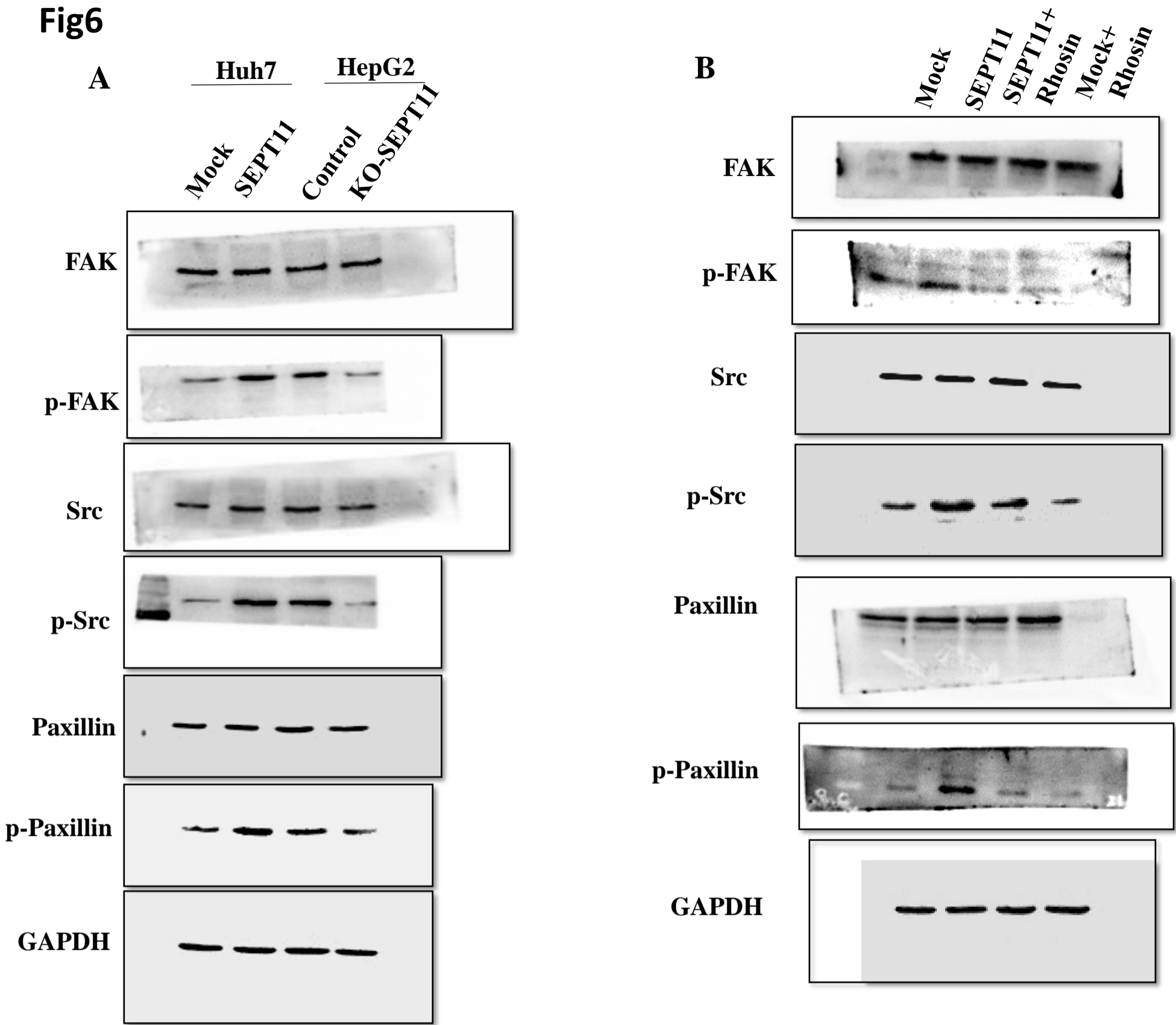

Fig6

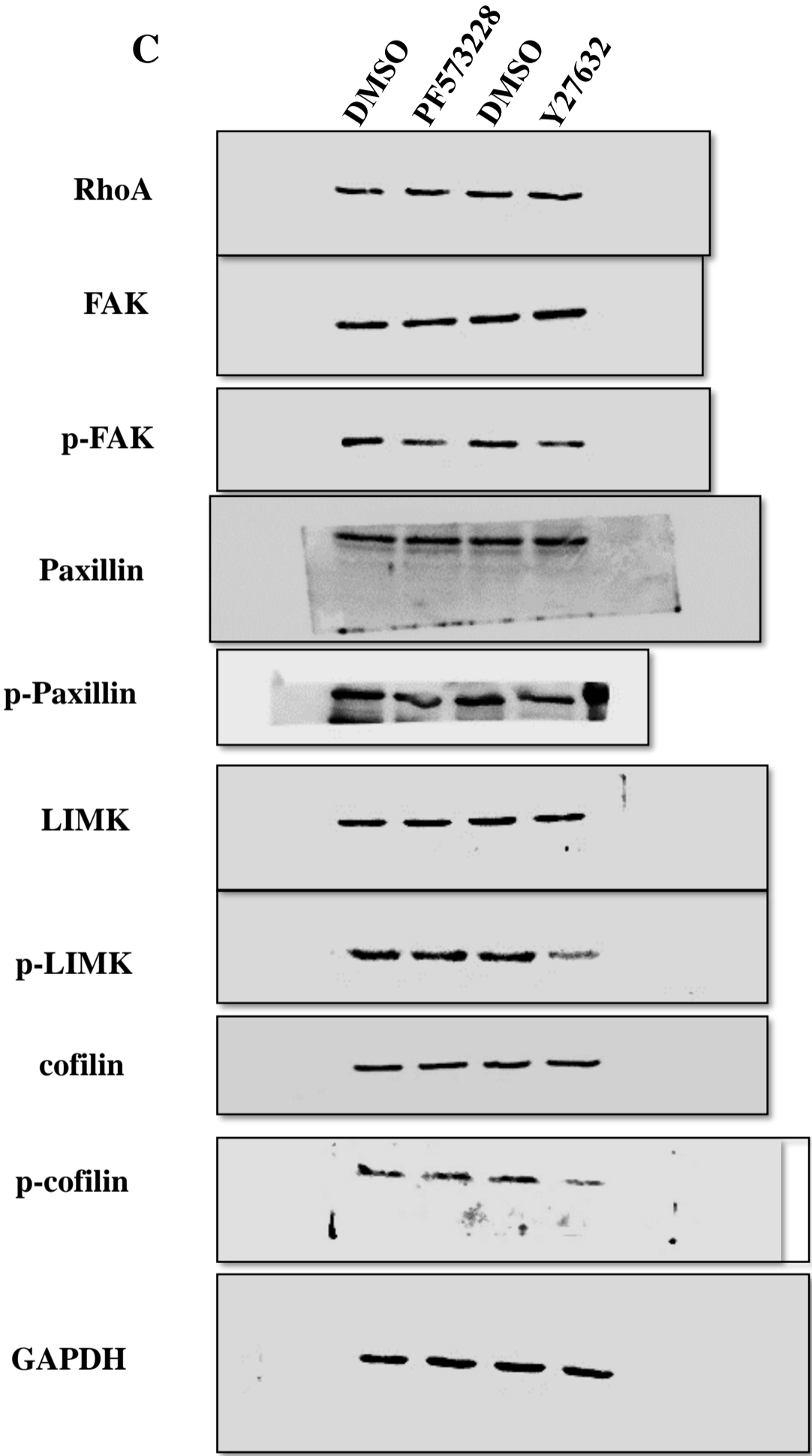

Fig7

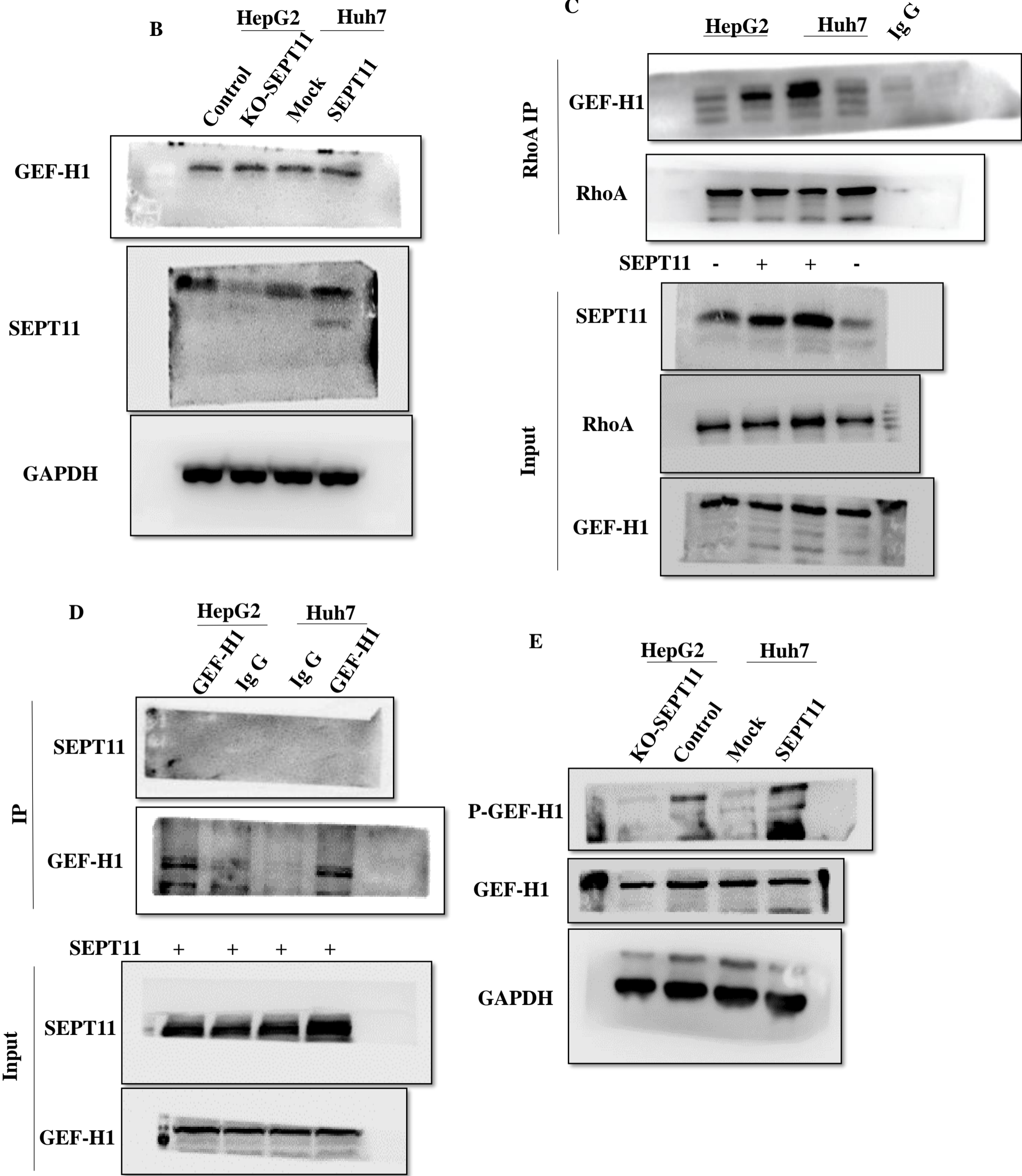

Fig7

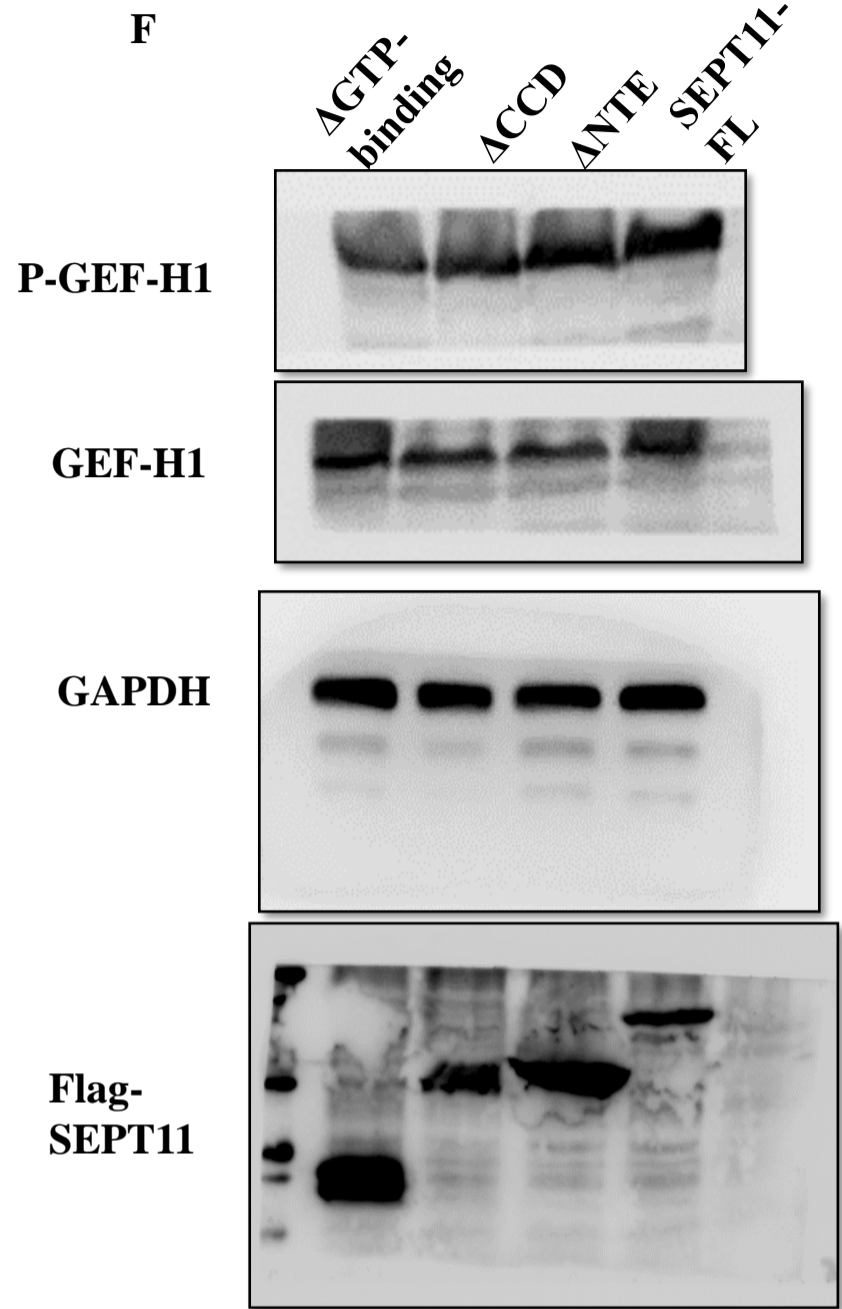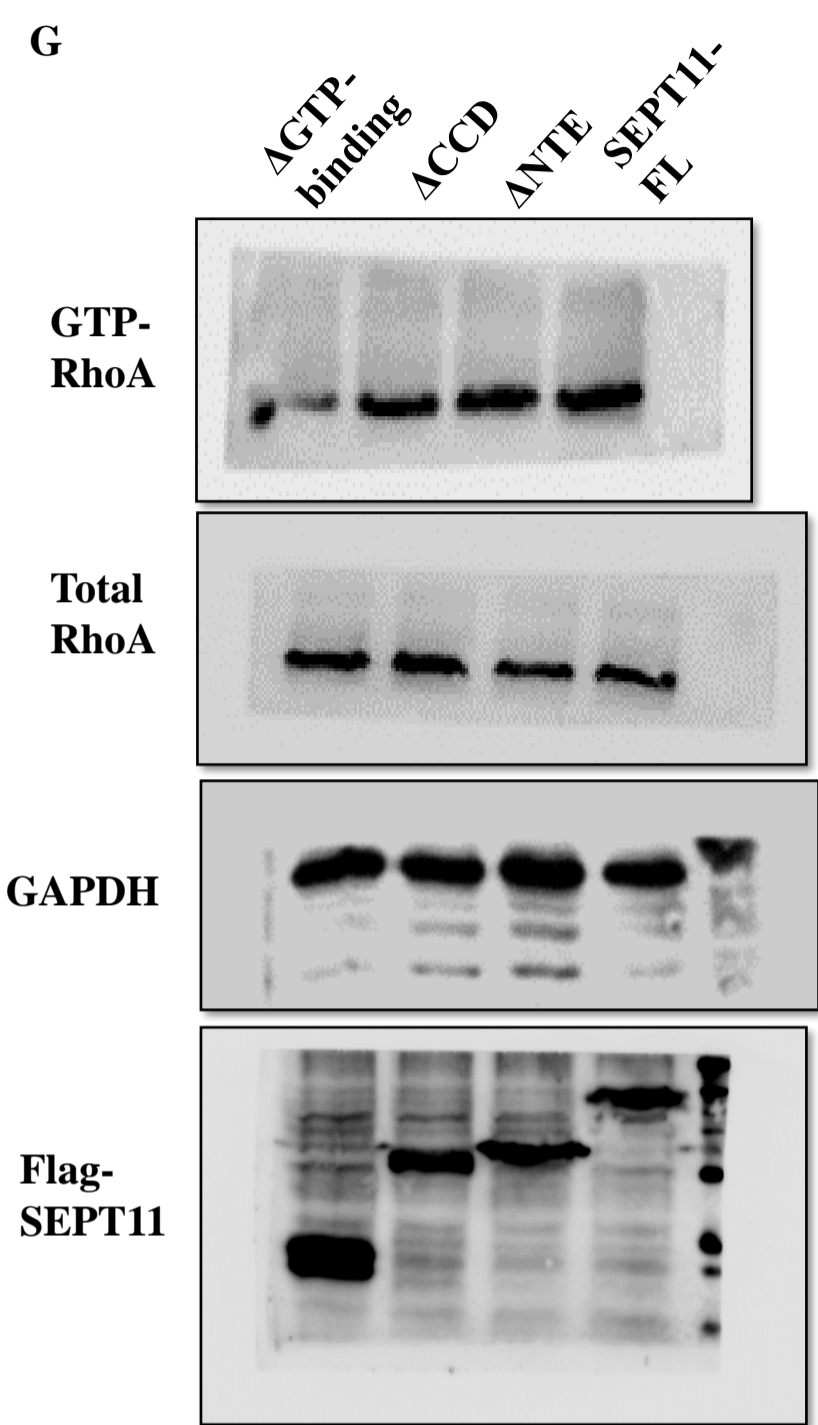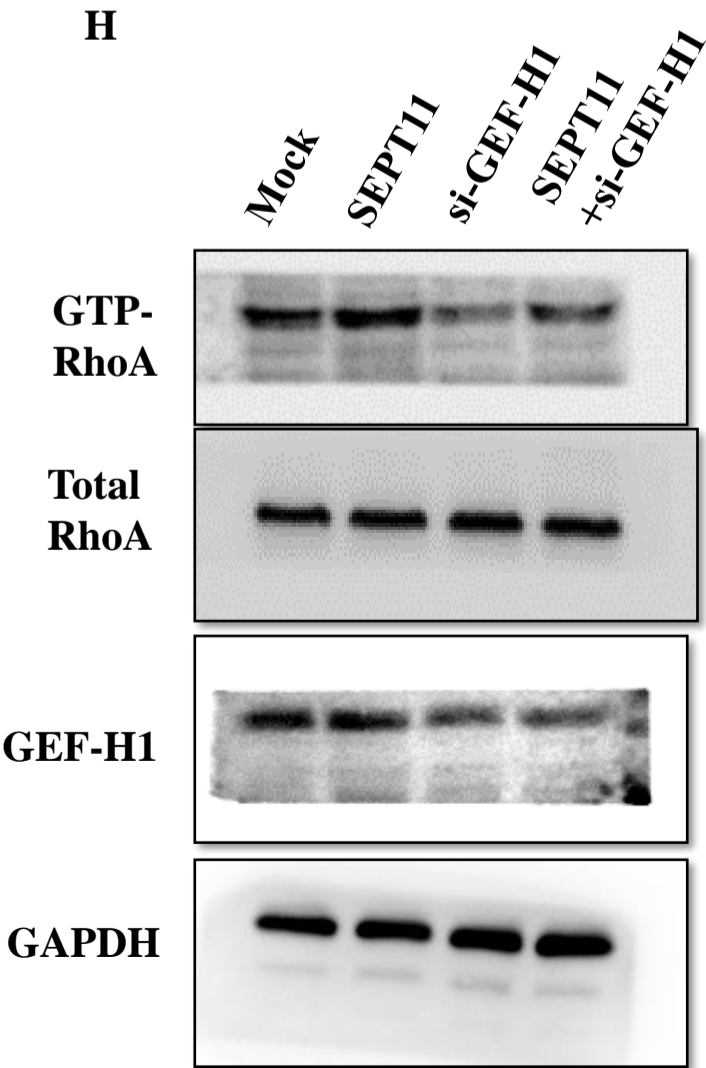

Supplement: Supplementary file 9 — Full and uncropped western blots [file 41419_2023_5726_MOESM9_ESM.pdf]
