# Supplementary material for: Septin11 promotes hepatocellular carcinoma cell motility by activating RhoA to regulate cytoskeleton and cell adhesion
Source: Cell Death Dis. 2023 Apr 20;14(4):280. doi: 10.1038/s41419-023-05726-y (PMC10119145; doi:10.1038/s41419-023-05726-y)
Supplement: Supplementary file 8 — Supplementary Figure legends [file 41419_2023_5726_MOESM8_ESM.docx]

**Supplementary Figure. 1 mRNA-seq and ATAC-seq to screen SEPT11 regulated by LncAY**

(A) volcanic map of significantly altered genes in AY overexpressed HepG2 cells; (B) volcanic map of genes with significantly open and closed sites in AY overexpressed HepG2 cells; (C) GO analysis of up-regulated genes in mRNA-seq; (D) KEGG analysis of up-regulated genes in mRNA-seq; (E-F) The effect of AY-related clones was verified in HepG2 and Huh7 cells, respectively; (G) The expression of AY and SEPT11 in 76 cases of HCC tissues.

**Supplementary Figure. 2 Bioinformatics analysis of SEPT11 expression in various tissues, tumor cell lines, cancer and paracancerous tissues**

(A) The expression levels of SEPT11 in 31 tissues were analyzed using GTEx database data; (B) SEPT11 gene expression in 21 tissue-derived tumor cell lines was analyzed using CCLE database data; (C) TCGA database was used to analyze the difference of SEPT11 between cancer and adjacent tissues in each tumor sample; (D) After integrating the data of normal tissue in the GTEx database and TCGA tumor tissue, the difference of SEPT11 expression between cancer and adjacent tissues in 27 kinds of tumors was analyzed; (E) Forest map of the relationship between SEPT11 expression and OS prognosis in 33 kinds of tumors was analyzed by TCGA data.

**Supplementary Figure. 3 Bioinformatics and experiments analyzed the function of SEPT11**

(A) GSEA analysis of HCC expression profiles in the TCGA database to analyze the TOP10 signaling pathway regulated by SEPT11; (B) QPCR to detect the effect of overexpressing SEPT11; (C) Colony formation assay detected the effect of SEPT11 on cell proliferation ability; (D) The effect of SEPT11 expression on proliferation-related factors was detected by WB; (E) subcutaneous tumorigenesis of Huh7-luciferase cells in nude mice was observed and photographed with mouse in vivo fluorescence imaging system on the 28th day; (F) subcutaneous tumorigenesis of HepG2 cells in nude mice was observed and photographed on the 28th day; (G)The lung tissues of mice with HepG2 cells tail vein were stained with CD31 antibody to observe angiogenesis.

**Supplementary Figure. 4 Regulation signal of SEPT11.**

(A-B) GSEA analysis of liver cancer expression profiles in the TCGA database, two other TOP5 signaling pathways related to invasion and migration regulated by SEPT11. (C) CO-IP and WB were used to detect the binding of SEPT11 and RhoA in HepG2 and Huh7 cells. (D) According to three domains of SEPT11: NTE (N-terminal extension), GTP binding (GTP binding domain), and CCD (Coiled coil domain), we have constructed exogenous plasmids with Flag tags. (E) CO-IP and WB were used to detect the binding of HA-RhoA to Flag-SEPT11 fragment and GEF-H1. (F) Transwell invasion assay effects of PF573228 on SEPT11-enhanced cell invasion.
