# Supplementary material for: Septin11 promotes hepatocellular carcinoma cell motility by activating RhoA to regulate cytoskeleton and cell adhesion
Source: Cell Death Dis. 2023 Apr 20;14(4):280. doi: 10.1038/s41419-023-05726-y (PMC10119145; doi:10.1038/s41419-023-05726-y)
Supplement: Supplementary file 10 — Cell STR Authentication [file 41419_2023_5726_MOESM10_ESM.pdf]

ZTKJ

细 胞 STR 分型检验报告

Report of Cell Line Identification

样本编号/ Sample No.： THLE-2

待检测细胞系名称/Name of cell line: THLE-2

样本数量及规格/ Sample Spec.： 细胞沉淀 1 个/ Cell precipitation

样本接收日期/ Sample Receive Date： 20210904

Experimental result

Genotype Test Results

Sample NO.	Multiple Alleles	Non - human Source Pollution	Matched Cell Line
01	(-)	(-)	THLE-2

STR Typing Result

Marker	STR Profile(**sample 01**)			
	Allele1	Allele2	Allele3	Allele4
D5S818	11,13			
	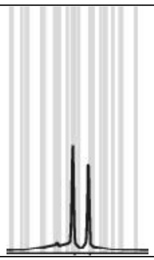			
D13S317	8,12			
	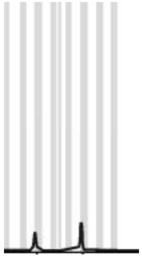			
D7S820	10,12			
	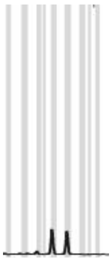			

ZTKJ

D16S539	11,13
	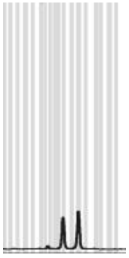
vWA	16,17
	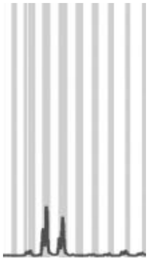
TH01	7,9.3
	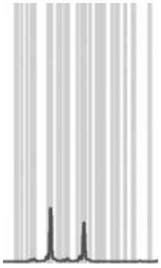
AMEL	X,Y
	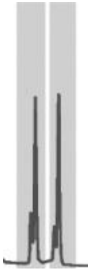
TPOX	8,11
	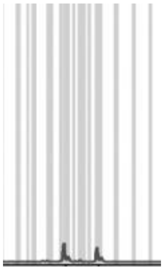
CSF1PO	11,13

ZTKJ

	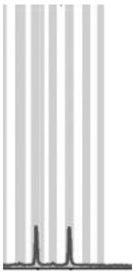

3 **Programme**

	Programme1	Programme2	Programme3	Programme4	Programme5
1	TH01	AMEL	TPOX	D3S1358	PENTAE
2	D12S319	D5S818	vWA	D13S317	
3	D7S820	D2S1338	D8S1179	D6S1043	
4	CSF1P0	D21S11		D16S539	
5	FGA	D18S51			

Laboratory Technician：Xin Wang

Review Member: Muyi Yang
