# Supplementary material for: Septin11 promotes hepatocellular carcinoma cell motility by activating RhoA to regulate cytoskeleton and cell adhesion
Source: Cell Death Dis. 2023 Apr 20;14(4):280. doi: 10.1038/s41419-023-05726-y (PMC10119145; doi:10.1038/s41419-023-05726-y)

Applied Biosystems

GeneMapper 4.1

1110-2

Sample File	Sample Name	Panel	Marker	AN	BD	DP	NB	CC	OVL	GQ
G1_Z1_A02.fsa	09	1Z	01-D5S818			NA	NA	NA	NA	

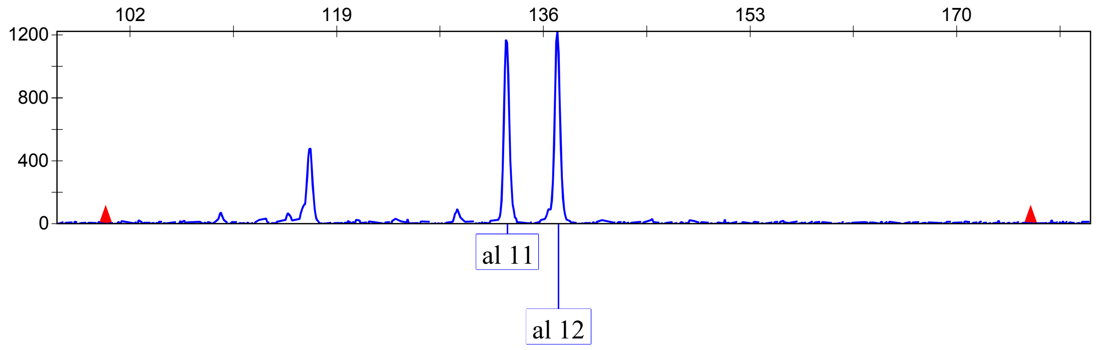

Sample File	Sample Name	Panel	Marker	AN	BD	DP	NB	CC	OVL	GQ
G1_Z2_A03.fsa	17	2Z	02-D13S317			NA	NA	NA	NA	

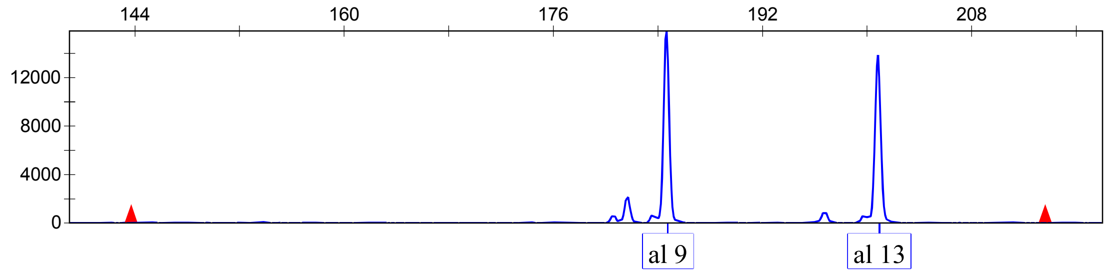

Sample File	Sample Name	Panel	Marker	AN	BD	DP	NB	CC	OVL	GQ
G1_Z1_A02.fsa	09	1Z	03-D7S820			NA	NA	NA	NA	

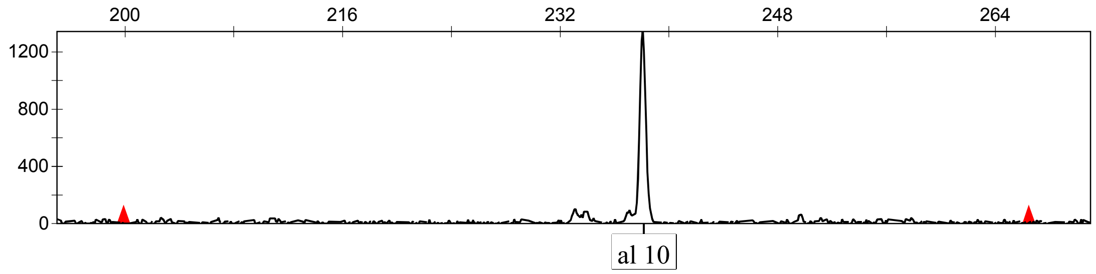

Sample File	Sample Name	Panel	Marker	AN	BD	DP	NB	CC	OVL	GQ
04_G1_A06.fsa	41	04-D16S539	04-D16S539			NA	NA	NA	NA	

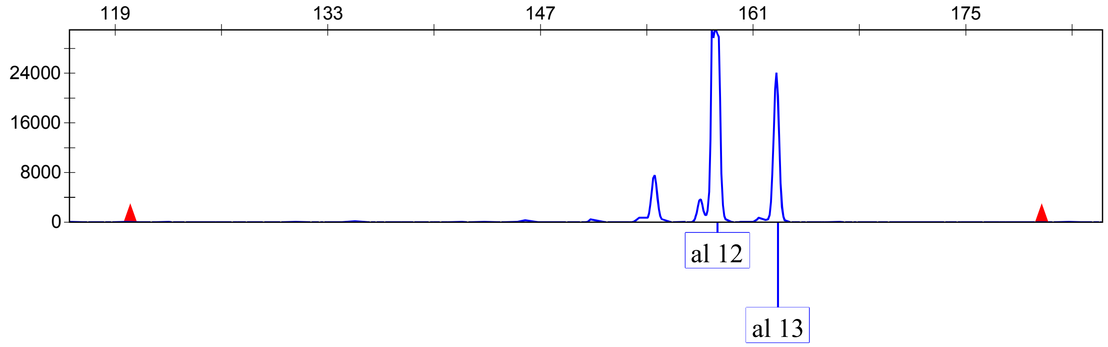

G1_Z1_A02.fsa	09	1Z	05-VWA	-4.0	-4.0	NA	NA	NA		

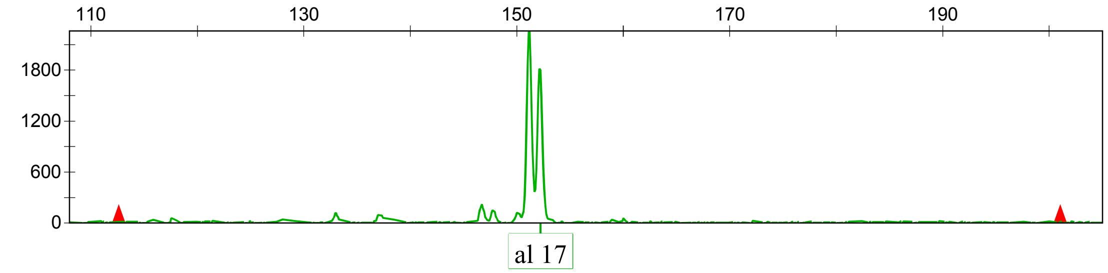

G1_Z2_A03.fsa	17	2Z	06-TH01			NA	NA	NA	NA	

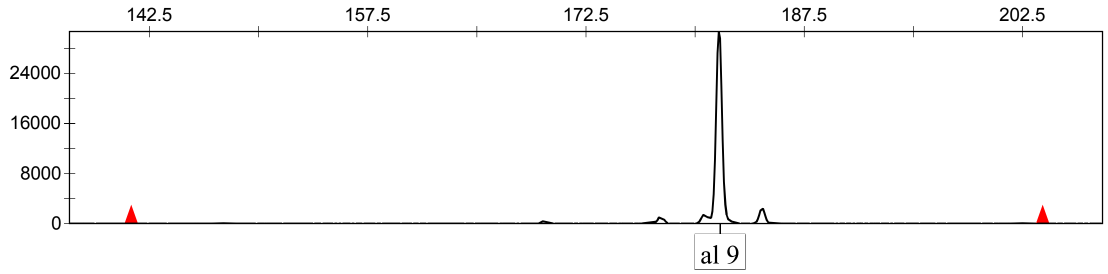

Sample File	Sample Name	Panel	Marker	AN	BD	DP	NB	CC	OVL	GQ
07_G1_A07.fsa	49	07-AMEL	07-AMEL			NA	NA	NA	NA	

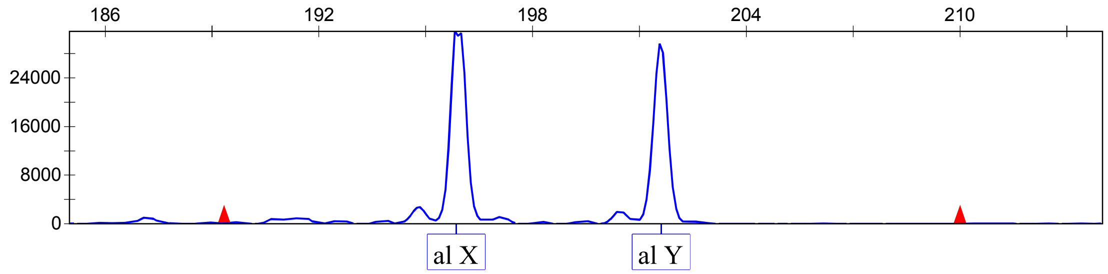

Sample File	Sample Name	Panel	Marker	AN	BD	DP	NB	CC	OVL	GQ
G1_Z1_A02.fsa	09	1Z	08-TOPX	-4.0	-4.0	NA	NA	NA		

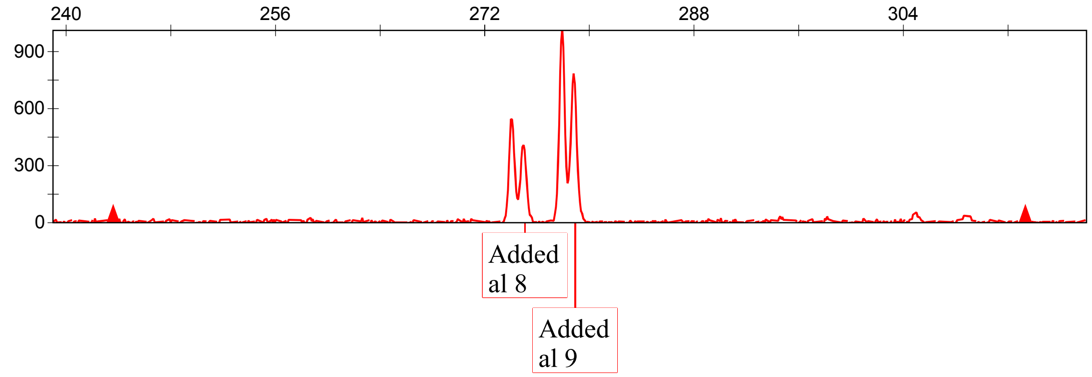

Sample File	Sample Name	Panel	Marker	AN	BD	DP	NB	CC	OVL	GQ
G1_Z2_A03.fsa	17	2Z	09-CSF1PO			NA	NA	NA	NA	

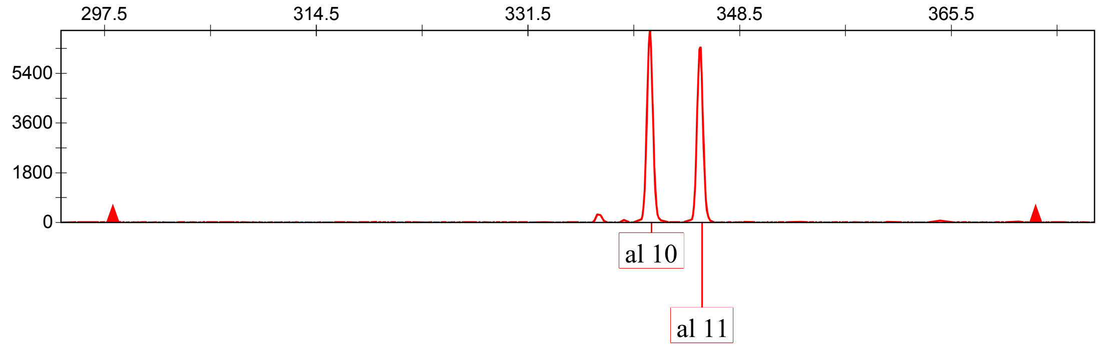

GeneMapper 4.1

Sample File	Sample Name	Panel	Marker	AN	BD	DP	NB	CC	OVL	GQ
G1_Z1_A02.fsa	09	1Z	10-D12S391			NA	NA	NA	NA	

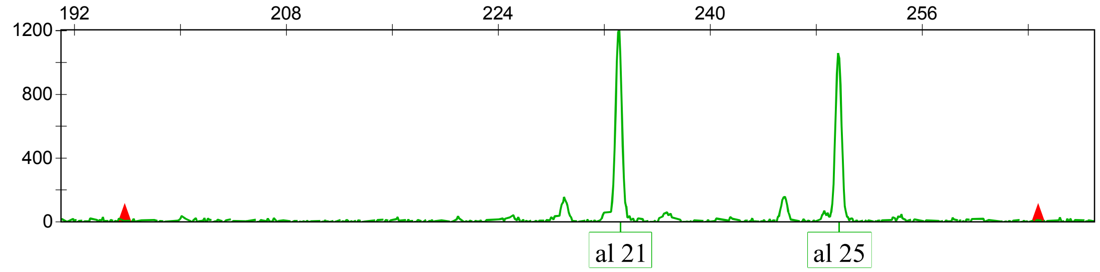

Sample File	Sample Name	Panel	Marker	AN	BD	DP	NB	CC	OVL	GQ
G1_Z2_A03.fsa	17	2Z	11-FGA			NA	NA	NA	NA	

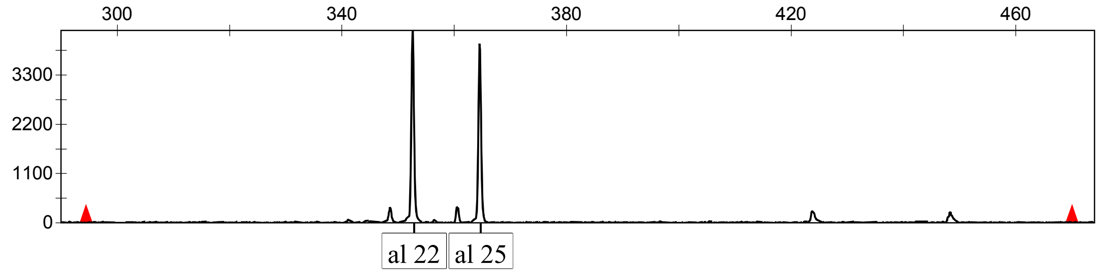

Sample File	Sample Name	Panel	Marker	AN	BD	DP	NB	CC	OVL	GQ
G1_Z2_A03.fsa	17	2Z	12-D2S1338			NA	NA	NA	NA	

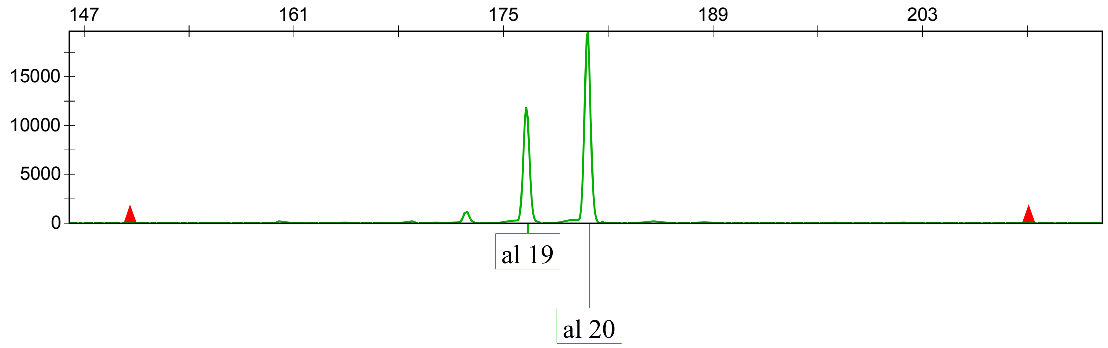

1110-2

Sample File	Sample Name	Panel	Marker	AN	BD	DP	NB	CC	OVL	GQ
G1_Z2_A03.fsa	17	2Z	13-D21S11	-4.0	-4.0	NA	NA	NA		

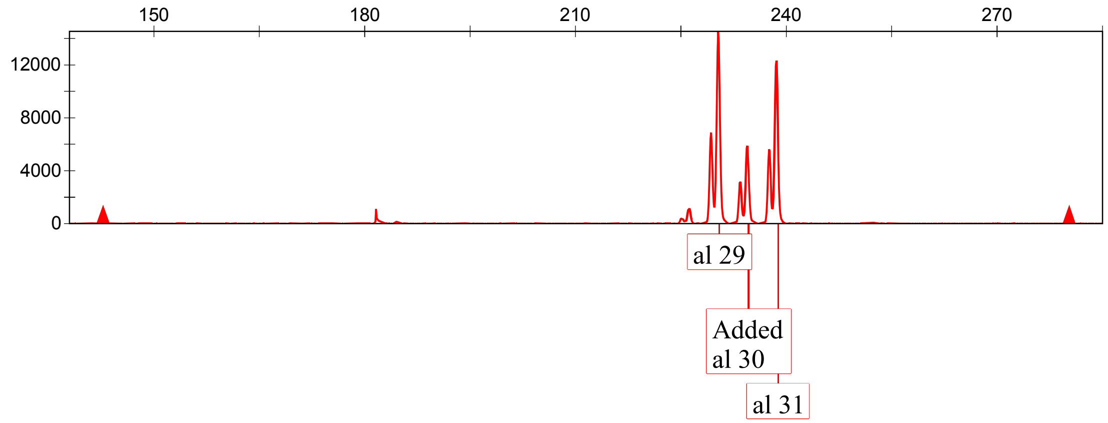

G1_Z1_A02.fsa	09	1Z	14-D18S51			NA	NA	NA	NA	

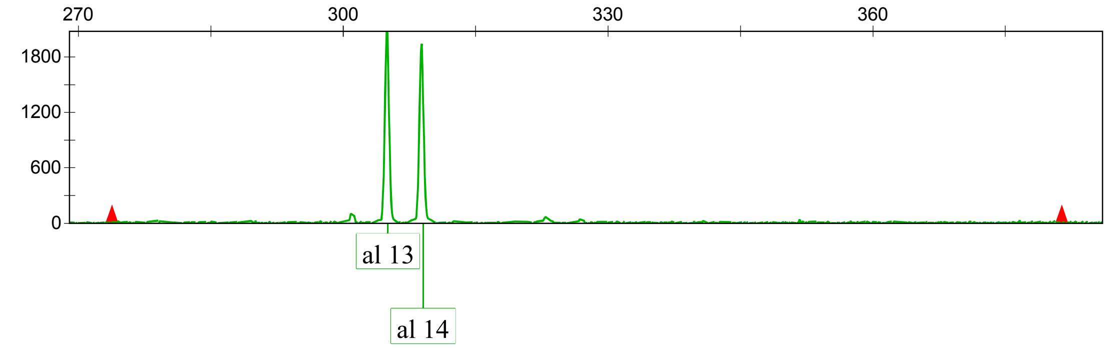

G1_Z1_A02.fsa	09	1Z	15-D8S1179			NA	NA	NA	NA	

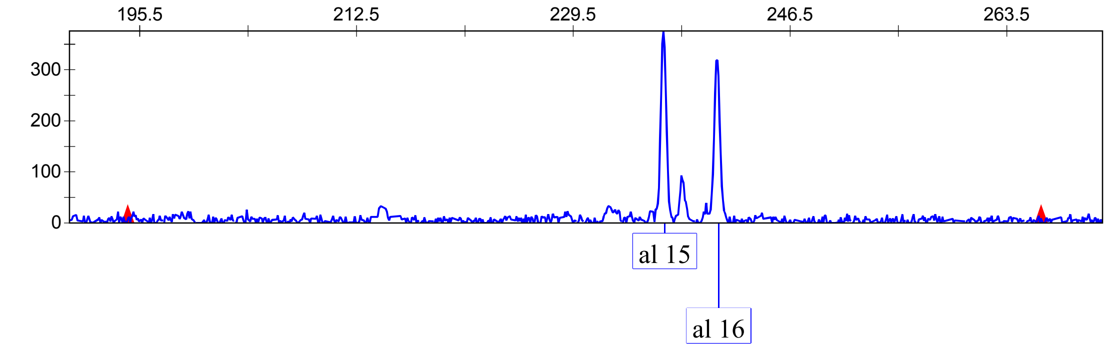

Sample File	Sample Name	Panel	Marker	AN	BD	DP	NB	CC	OVL	GQ
G1_Z1_A02.fsa	09	1Z	16-D3S1358		-4.0	NA	NA	NA		

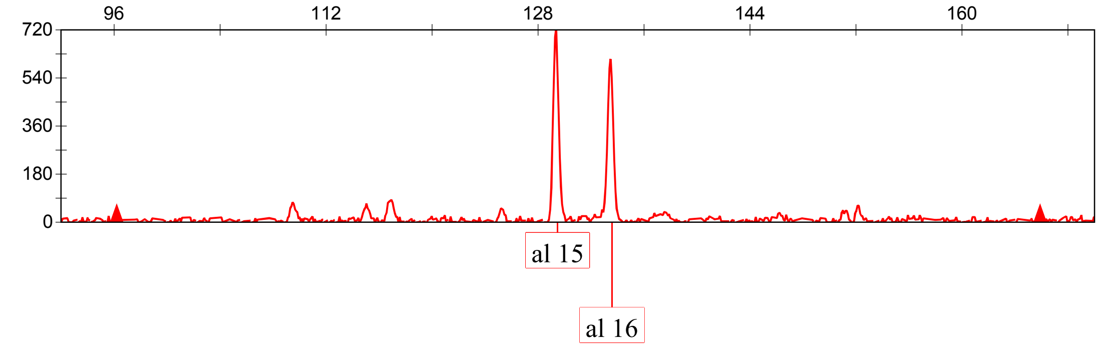

17_G1_A10.fsa	73	17-D6S1043	17-D6S1043			NA	NA	NA	NA	

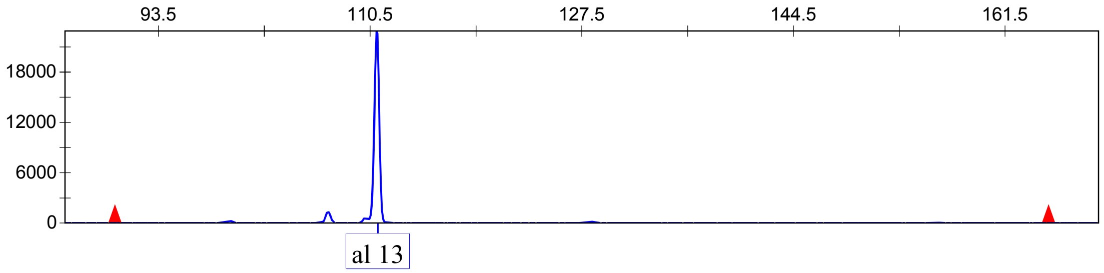

G1_Z2_A03.fsa	17	2Z	18-PENTAE			NA	NA	NA	NA	

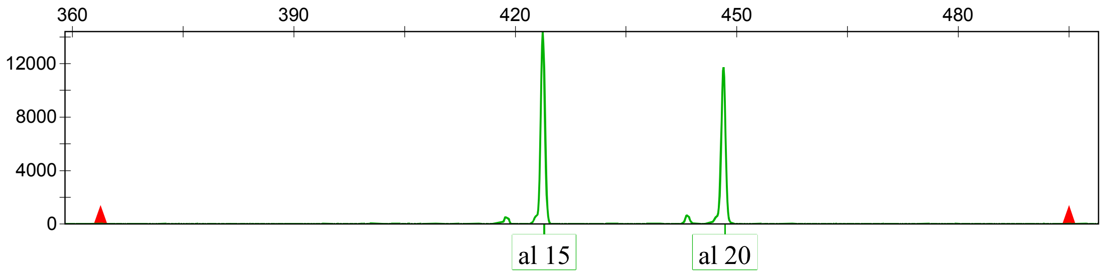

Sample File	Sample Name	Panel	Marker	AN	BD	DP	NB	CC	OVL	GQ
G1 Z1 A02.fsa	09	1Z	19-D19S433			NA	NA	NA	NA	

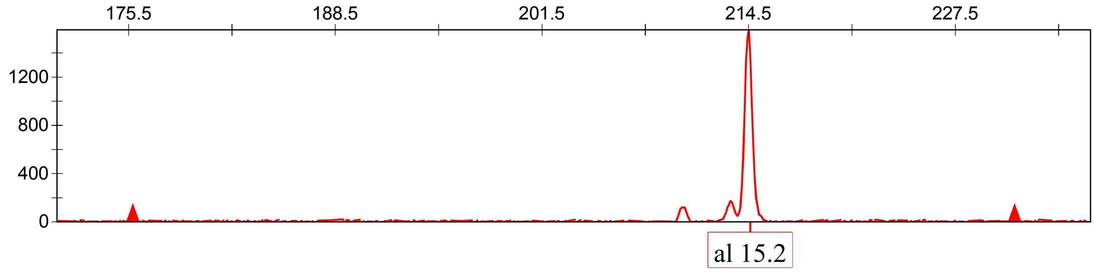

Sample File	Sample Name	Panel	Marker	AN	BD	DP	NB	CC	OVL	GQ
G1 Z2 A03.fsa	17	2Z	20-PENTAD			NA	NA	NA	NA	

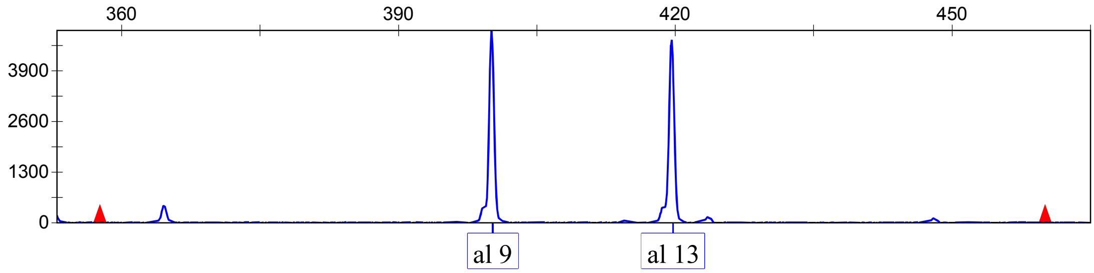

Supplement: Supplementary file 11 — Cell STR Authentication [file 41419_2023_5726_MOESM11_ESM.pdf]
