# Supplementary material for: Septin11 promotes hepatocellular carcinoma cell motility by activating RhoA to regulate cytoskeleton and cell adhesion
Source: Cell Death Dis. 2023 Apr 20;14(4):280. doi: 10.1038/s41419-023-05726-y (PMC10119145; doi:10.1038/s41419-023-05726-y)

Applied Biosystems
GeneMapper ID v3.2

20210930(2)

Sample File	Sample Name	Panel	SQO	OS	SQ
20_D03_CellLineAuthentication-2-1008.fsa	293T	21Plex_STR_Panel_v1.1			

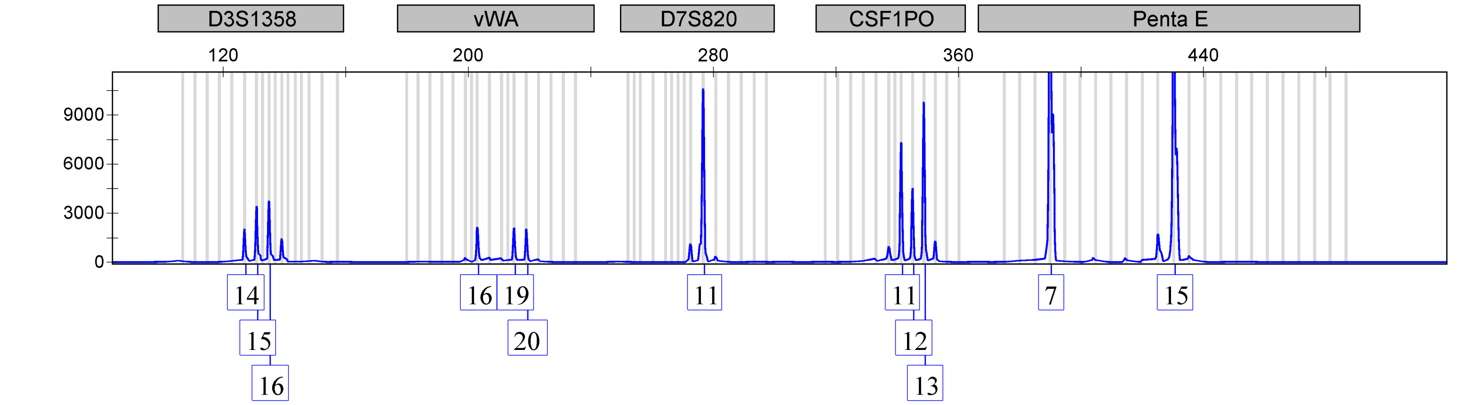

20_D03_CellLineAuthentication-2-1008.fsa	293T	21Plex_STR_Panel_v1.1			

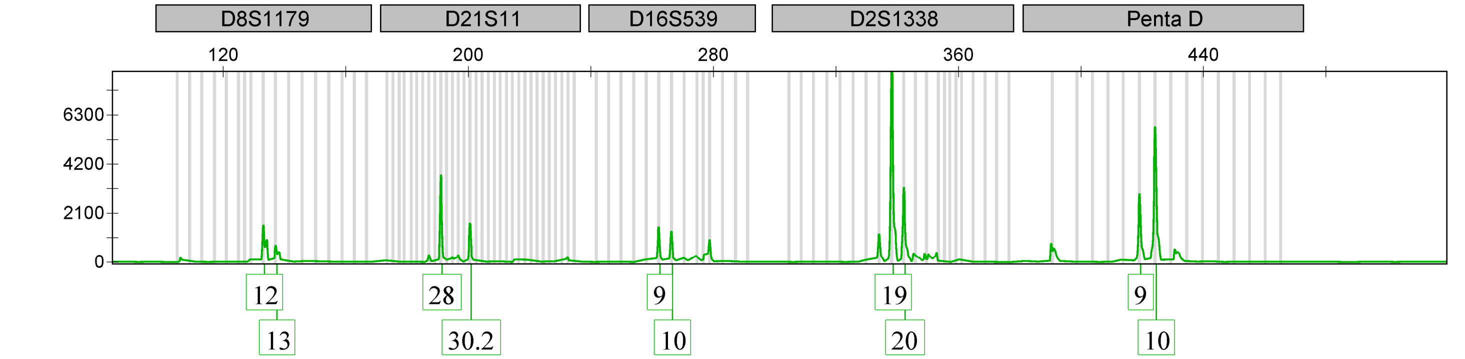

20_D03_CellLineAuthentication-2-1008.fsa	293T	21Plex_STR_Panel_v1.1			

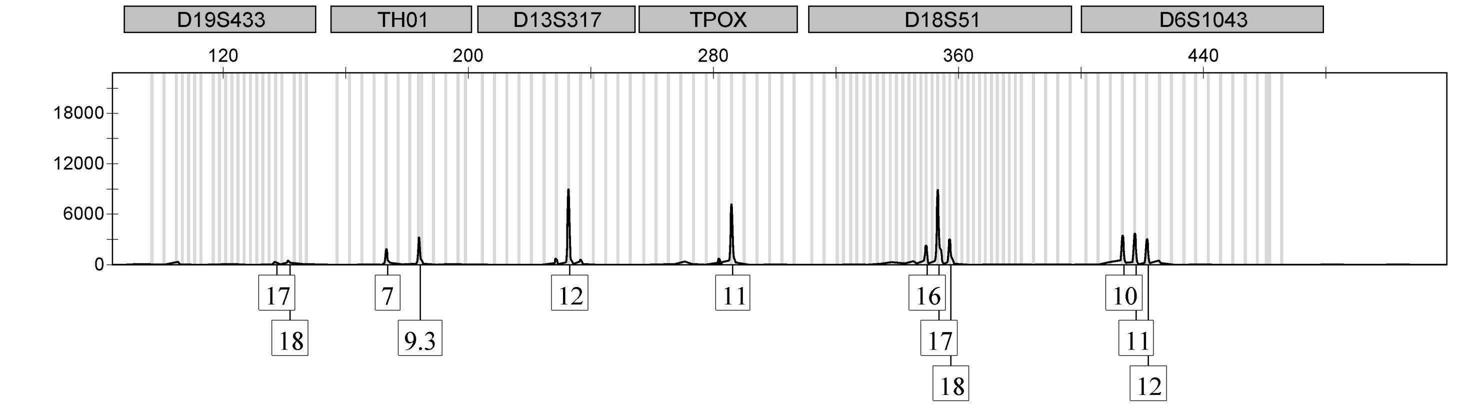

Applied Biosystems

GeneMapper ID v3.2

20210930(2)

Sample File	Sample Name	Panel	SQO	OS	SQ
20_D03_CellLineAuthentication-2-1008.fsa	293T	21Plex_STR_Panel_v1.1			

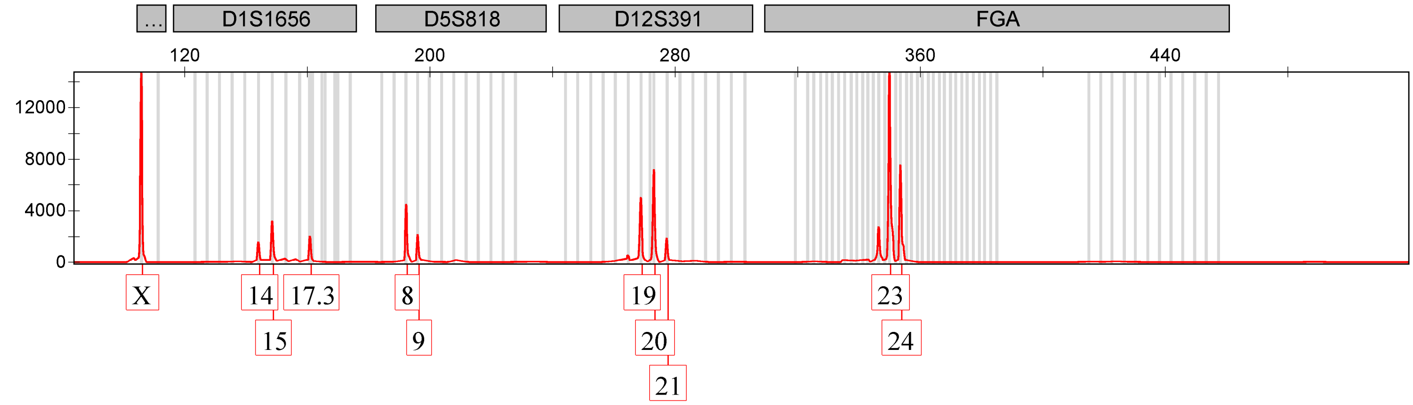

Supplement: Supplementary file 12 — 293T STR [file 41419_2023_5726_MOESM12_ESM.pdf]
