# Supplementary material for: Septin11 promotes hepatocellular carcinoma cell motility by activating RhoA to regulate cytoskeleton and cell adhesion
Source: Cell Death Dis. 2023 Apr 20;14(4):280. doi: 10.1038/s41419-023-05726-y (PMC10119145; doi:10.1038/s41419-023-05726-y)

20210930(2)

Sample File	Sample Name	Panel	SQO	OS	SQ
21_E03_CellLineAuthentication-2-1008.fsa	Huh7	21Plex_STR_Panel_v1.1			

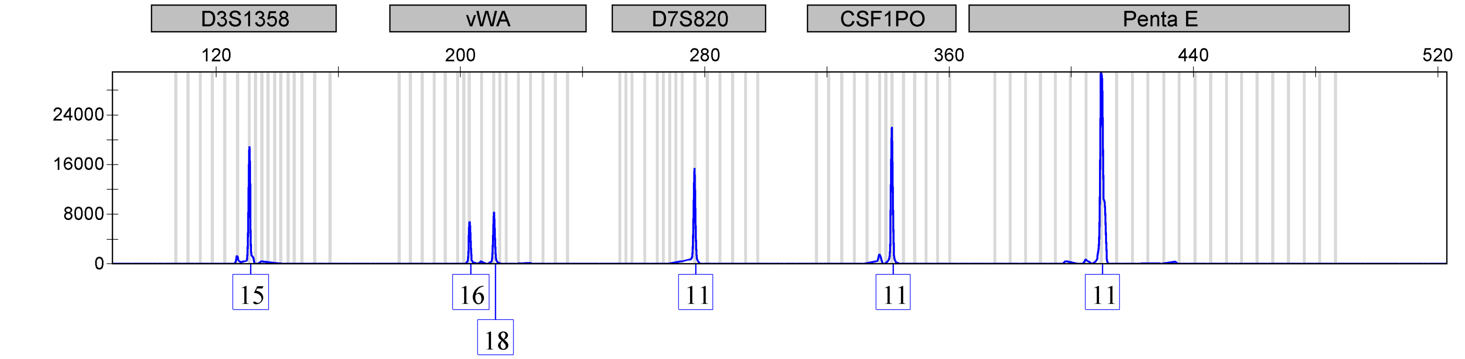

Sample File	Sample Name	Panel	SQO	OS	SQ
21_E03_CellLineAuthentication-2-1008.fsa	Huh7	21Plex_STR_Panel_v1.1			

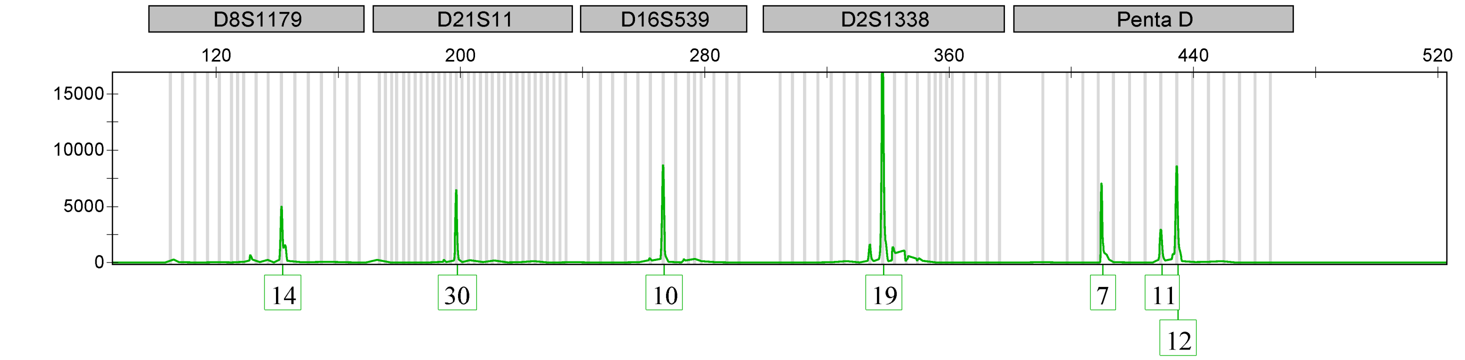

Sample File	Sample Name	Panel	SQO	OS	SQ
21_E03_CellLineAuthentication-2-1008.fsa	Huh7	21Plex_STR_Panel_v1.1			

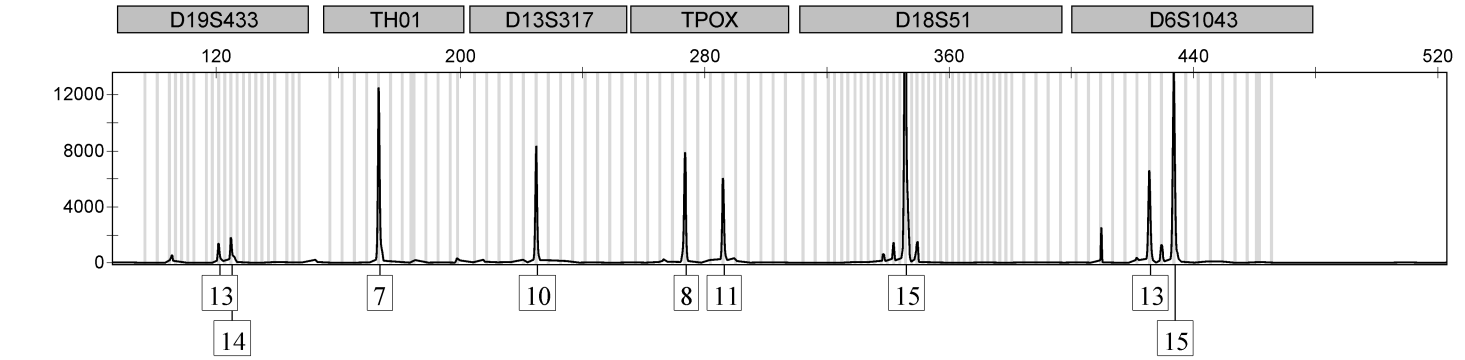

Sample File	Sample Name	Panel	SQO	OS	SQ
21_E03_CellLineAuthentication-2-1008.fsa	Huh7	21Plex_STR_Panel_v1.1			

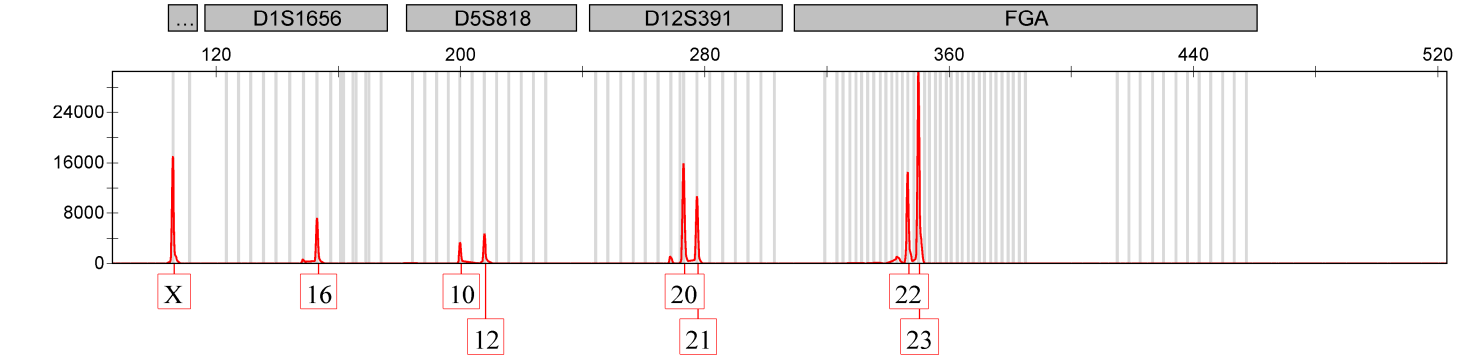

Supplement: Supplementary file 13 — Cell STR Authentication [file 41419_2023_5726_MOESM13_ESM.pdf]
